# De Novo Long-Read Whole-Genome Assemblies and the Comparative Pan-Genome Analysis of Ascochyta Blight Pathogens Affecting Field Pea

**DOI:** 10.3390/jof8080884

**Published:** 2022-08-22

**Authors:** Yvonne O. Ogaji, Robert C. Lee, Tim I. Sawbridge, Benjamin G. Cocks, Hans D. Daetwyler, Sukhjiwan Kaur

**Affiliations:** 1 Agriculture Victoria, AgriBio, Centre for AgriBioscience, 5 Ring Road, Melbourne, VIC 3083, Australia; 2 School of Applied Systems Biology, La Trobe University, Melbourne, VIC 3086, Australia; 3 Centre for Crop and Disease Management, School of Molecular and Life Sciences, Curtin University, Perth, WA 6102, Australia

**Keywords:** nuclear genome, mitochondrial genome, CAZymes, orthologs, comparative genomics

## Abstract

Ascochyta Blight (AB) is a major disease of many cool-season legumes globally. In field pea, three fungal pathogens have been identified to be responsible for this disease in Australia, namely *Peyronellaea pinodes*, *Peyronellaea pinodella* and *Phoma koolunga*. Limited genomic resources for these pathogens have been generated, which has hampered the implementation of effective management strategies and breeding for resistant cultivars. Using Oxford Nanopore long-read sequencing, we report the first high-quality, fully annotated, near-chromosome-level nuclear and mitochondrial genome assemblies for 18 isolates from the Australian AB complex. Comparative genome analysis was performed to elucidate the differences and similarities between species and isolates using phylogenetic relationships and functional diversity. Our data indicated that *P. pinodella* and *P. koolunga* are heterothallic, while *P. pinodes* is homothallic. More homology and orthologous gene clusters are shared between *P. pinodes* and *P. pinodella* compared to *P. koolunga*. The analysis of the repetitive DNA content showed differences in the transposable repeat composition in the genomes and their expression in the transcriptomes. Significant repeat expansion in *P. koolunga’s* genome was seen, with strong repeat-induced point mutation (RIP) activity being evident. Phylogenetic analysis revealed that genetic diversity can be exploited for species marker development. This study provided the much-needed genetic resources and characterization of the AB species to further drive research in key areas such as disease epidemiology and host–pathogen interactions.

## 1. Introduction

Ascochyta Blight (AB), also referred to as black spot, is a devastating disease of many cool-season legumes of commercial importance, including field pea, lentil, chickpea, and faba bean. AB, caused by fungal species from the phylum Ascomycota, is a foliar disease affecting all of the above-ground organs of the plant, including the stem, leaves, pods, and flowers. AB affects most legume growing regions around the world, especially Europe, Australia, New Zealand, and North America. It is a major production constraint in all of the areas where these crops are produced, presenting significant crop losses as well as a reduction in yield and grain quality [1,2,3].

Unlike other legumes where only one ascochyta species is responsible for the disease, there are at least seven reported species which are responsible for AB in field peas, including: *Ascochyta pisi* Lib., *Peyronellaea pinodes* (Berk. and A. Bloxham) (Synonyms: *Ascochyta pinodes, Mycosphaerella pinodes*, Teleomorph: *Didymella pinodes*), *Peyronellaea pinodella* (L.K. Jones) (Synonym: *Ascochyta pinodella*, syn. *Phoma medicaginis var. pinodella*), *Phoma koolunga, Phoma herbarum, Boerma exigua var. exigua,* and *Phoma glomerata* [4,5,6,7,8,9,10]. Significant yield losses have been reported across the world, although varying environmental conditions and cropping systems affect the level of damage due to ascochyta blight. Up to 50% losses have been reported in Canada, 40% in France, 53% in Ethiopia, and 30% in China; a disease severity of 72% has been reported in Spain [11,12,13,14,15]. In Australia, AB is the second most important biotic stress affecting field pea production after bacterial blight caused by *Pseudomonas syringae*. Average annual losses to AB are from 10 to 60% in Australia, with higher losses observed when environmental conditions such as rain and wind favour disease progression [16,17]. Economic losses can occur through a reduction in crop biomass or the infection of pods and seeds that makes the grain unmarketable [18,19,20,21,22].

In Australia, AB presents as a complex of mainly *P. pinodes*; *P. pinodella* and *P. koolunga* in varying proportions, with *P. pinodella* seemingly being the least prevalent. With three causal pathogen species, the management of AB is challenging and complex, and requires a combination of management strategies [16,20,23,24]. The frequency of individual pathogen species varies within the growing season, further limiting the effectiveness of more generalized management approaches. The most efficient management system is the use of resistant cultivars; however, due to the multi-species disease complex, breeding for AB resistance has been difficult [16,25]. Resistance to one species has no significant impact on crop and yield loss because this leads to the increased prevalence of the other AB complex species infecting the crop.

The characterization of AB species in chickpea and lentil is well advanced [26,27,28]; nonetheless, there is a gap in the knowledge of the AB pathogen complex in field pea. The morphological characterization of AB species can vary significantly, and is largely dependent on culturing techniques; the similar colony morphology for different species also makes it challenging to assign isolates to species [29]. Extensive genetic characterization of the AB complex is lacking, and in addition, *P. pinodes* and *P. pinodella* are not easily distinguishable using current molecular methods due to their high genomic similarity. *P. koolunga*, discovered in 2009, seems to be more taxonomically different from *P. pinodes* and *P. pinodella,* [29,30,31,32]. Whole-genome assemblies are required in order to characterize the different AB species, understand host–pathogen interactions, and enable genomically assisted breeding for the development of resistant varieties. To date, no whole genome sequence assembly of the field pea Ascochyta species has been published, although there is a published mitochondrial genome for *M. pinodes* [33].

Whole-genome information is vital for the development of reliable field diagnostic tests that can accurately identify different AB species that can co-exist on the crop. This will advance our understanding of the disease epidemiology [6,29] and help to define appropriate management strategies. Research reveals that sexual reproduction and host genetic diversity contribute to virulence; therefore, the determination of the mating type for the AB species—especially for *P. koolunga,* the mating type of which has not yet been determined—could drive further research [16,34]. Understanding host–pathogen interactions is also crucial for the elucidation of the primary features of the plant immune response. These findings can unravel the genetic mechanisms underlying plant defenses and disease resistance, which facilitates functional marker development and application in crop breeding programs. Fungal whole-genome assemblies can help with the eludication of host–pathogen interactions because fungal genome plasticity and transposable elements enable them to evolve and adapt at a rapid rate, making it challenging to develop suitable management strategies to aid plant protection [35,36,37]. Thus, an in-depth genetic characterization of the main AB complex species is an important first step towards finding better ways to control disease outbreaks, develop targeted and effective fungicides, and advance the development of AB-resistant field pea varieties.

One approach to better understand the genetic diversity and gene distribution within species is to combine comparative genomics with pan-genome analysis. Comparative genomics compares the genomic content of one species to another at micro- as well as macro-syntenic levels, based on gene annotation, the characterization of genome plasticity, and other evolutionary events, and resolves phylogenetic relationships [38,39,40,41]. While comparative genomic analysis can span kingdoms, pan-genome analysis was developed to narrow down the understanding of similarities and differences between strains or isolates of the same species or genus [42,43,44,45,46]. Pan-genome analysis enables the investigation of the functional diversity and abundance of genes, as well as the core and accessory gene distribution between species. Genome-based studies have shown that lineage-specific accessory genes can encode virulence determinants (avirulence genes), and the identification of such genes can enable targeted breeding for the improvement of plant resistance genes in order to prevent disease [47,48,49,50].

Advances in next-generation sequencing technologies (NGS) have enabled the sequencing of many non-model organisms at a reduced cost. This provides genetic resources that are useful for comprehensive comparative analysis. NGS includes short-read sequencing technologies such as Illumina and long-read sequencing platforms such as Oxford Nanopore Technologies (ONT) [51,52,53]. The assembly methods for short-read sequencing lack the resolution and capability to account for structural variants and sequence repeats, which are major features of complex eukaryotes such as fungi. Biodiversity research using molecular markers that exceed 550 bp for greater taxonomic resolution is also significantly impeded [54,55,56,57]. In contrast, long-read sequencing technologies resolves all of these drawbacks through individual reads long enough to cover entire repeat sequences. and the bioinformatic tools available for error correction and polishing can help to resolve their high error rate [58,59,60,61,62,63,64]. Consequently, species and genotype-level taxonomic resolutions have been greatly improved using long-read sequencing in fungi, which in turn, could improve disease monitoring strategies, epidemiological studies and diagnostic tool development [65,66].

To the best of our knowledge, no genome assembly is available for these three AB species to date. Using long- and short-read sequencing technology, the aim of this study was to obtain high-quality genome assemblies that will play an important role in future research to understand these species and how they interact with their host plant and the environment. Here, we present the first fully annotated nuclear and mitochondrial draft genome assemblies for 18 AB isolates, six each of *P. pinodes*, *P. pinodella* and *P. koolunga*. Our investigations included comparative and pan-genome analysis, genome annotation, and the exploration of secondary metabolites, CAZymes, orthologs and phylogenetic relationships. We have generated a valuable resource.

## 2. Materials and Methods

### 2.1. Fungal Culture

Eighteen field pea AB isolates were obtained from the Ascochyta pathogen culture collection at the Centre for Crop and Disease Management, Curtin University, Western Australia. The isolates were received as dried cultures on filter paper preserved with desiccants in airlocked bags. Six isolates for each species were used for this study. These included *P. pinodes* (Isolate3Pp; Isolate4Pp; Isolate5Pp; Isolate87Pp; Isolate88Pp; Isolate97Pp), *P. pinodella* (Isolate18Ppll; Isolate27Ppll; Isolate72Ppll; Isolate58Ppll; Isolate104Ppll; Isolate113Ppll) and *P. koolunga* (Isolate1Pk; Isolate2Pk; Isolate22Pk; Isolate32Pk; Isolate36Pk; Isolate42Pk). The isolates were cultured on potato dextrose agar (Difco laboratories, USA) and grown in a temperature- and light-controlled incubator for 5–15 days with a photoperiod of 12 h/23 °C dark and 12 h/19 °C black light (a UV-A fluorescent tube). The mycelial plugs were cut from media plates and grown in potato dextrose broth for 3–4 days at 22 °C in an incubator shaker at 150 rpm in the dark.

### 2.2. Nucleic Acid Extraction

For the DNA extraction, mycelial samples were harvested and freeze dried for 24 h. The total DNA was isolated from the freeze-dried samples using the Wizard^®^ Genomic DNA Purification System (Promega, Madison, WI, USA) as per the manufacturer’s instructions to achieve high-molecular-weight DNA. The eluted DNA was quantified using a Qubit^TM^ dsDNA BR Assay Kit (Thermo Fisher Scientific, Waltham, MA, USA), and the quality was assessed using a Nanodrop 2000 (Thermo Fisher Scientific). The integrity and molecular weight were assessed using an Agilent TapeStation 2200 system (Agilent Technologies, Santa Clara, CA, USA) following manufacturer’s instructions.

For RNA extraction, mycelial samples were collected into 1.5 mL Eppendorf tubes and stored at −80 °C until they were processed. The RNA extraction was carried out using a Plant/Fungi Total RNA Purification Kit (Norgen Biotek Corp., Thorold, ON, Canada). Quality was assessed using a Nanodrop 2000 spectrophotometer (Thermo Fisher Scientific), and integrity was assessed using an Agilent TapeStation 2200 system.

### 2.3. Oxford Nanopore Technologies (ONT) Sequencing

All of the ONT flow cells, library preparation kits and other consumables were sourced from Oxford Nanopore Technology (Oxford, England). The genome libraries for the isolates *P. pinodes* (Isolate3Pp; Isolate4Pp; Isolate5Pp), *P. pinodella* (Isolate18Ppll) and *P. koolunga* (Isolate1Pk; Isolate2Pk) were prepared using the ONT 1D ligation sequencing kit (SQK-LSK109) for MinION, with no shearing. For the remaining isolates, genome libraries were prepared using the SQK-LSK109 native barcode genomic DNA protocol for PromethION. The libraries were prepared according to the manufacturer’s instructions; however, the loading concentrations were adjusted to achieve the optimum loading of the flow cell.

Six high-molecular-weight ONT libraries with sizes ranging from 20 Kb to approximately 60 Kb for Isolate1Pk, Isolate2Pk, Isolate3Pp, Isolate4Pp, Isolate5Pp and Isolate18Ppll were sequenced using the ONT MinION platform. Each library was sequenced on one R9.4 flow cell (FLO-MIN106, ONT), except for Isolate2Pk, which was sequenced on two R9.4 flow cells, and the reads were combined. Additional sequencing was undertaken with all 18 high-molecular-weight ONT libraries, with sample sizes ranging from 10 Kb to approximately 60 Kb being sequenced using the ONT PromethION platform. Each library was sequenced on a PromethIon flow cell (FLO-PRO002, ONT). Fastq data from MinION and PromethION for each isolate were combined before proceeding to further analysis.

### 2.4. Transcriptome Sequencing and Alignment

**Figure 1:**
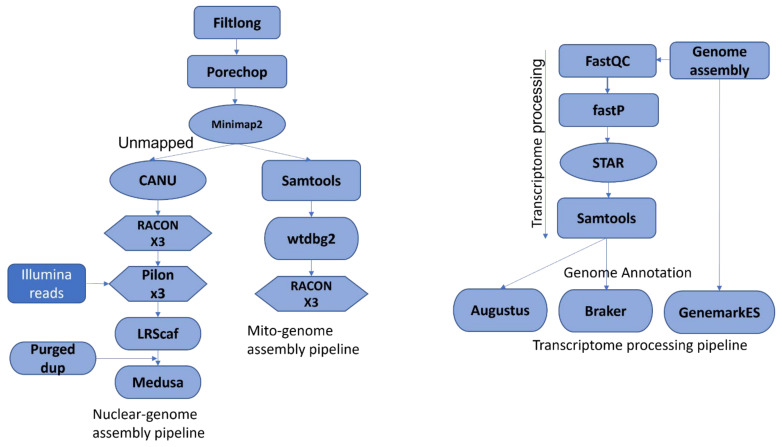
Assembly and annotation pipelines for nuclear, mitochondrial, and RNAseq data.

RNA samples extracted as detailed above were used to prepare transcriptome libraries for all 18 isolates in triplicate using Agilent’s Sure Select Strand Specific RNA Library Preparation Kit (Agilent Technologies) according to the manufacturer’s protocol. The RNA concentrations were estimated by running a sample of each library on an Illumina MiSeq sequencer (Illumina, Inc., San Diego, CA, USA) to measure library-specific barcode concentrations, and were pooled to achieve even proportions of each library in the pooled sample. Paired end sequencing (151 cycles + 151 cycles) was carried out on a NovaSeq 3000 (Illumina, Inc., San Diego, CA, USA). The RNA-seq data were processed according to the pipeline shown in Figure 1. FastQC v0.11.8 (http://www.bioinformatics.babraham.ac.uk/projects/fastqc/ was used to check the quality of the sequencing data. Fastp v0.21.0 [67] was used to trim the adaptors. STAR v2.7.3 [68] was used to align the processed transcripts to the respective genomes generating transcript alignment files in BAM format to be used for genome annotation.

### 2.5. Repeat Expression Analysis

The results from RepeatMasker for all of the isolates in GFF3 format were used to extract repeat sequences from the assembled AB genomes with getfasta in bedtools [69]. Repeat fasta files were separated by species and concatenated into a single file per species before clustering to obtain only the unique repeats present in each species using CDHITS [70]. The transcripts were quantified using Salmon [71] to obtain the number of reads for each repeat element.

### 2.6. Nuclear and Mitochondrial Genome Assembly, and Post Assembly Analysis

Sequencing data from MinION and PromethION was acquired using MinKNOW v0.51.1.62 (Oxford Nanopore Technologies. Oxford, United Kingdom). Raw fastq reads were processed as shown in Figure 1 and were categorized into “pass” or “fail” based on an average quality score > Q7. Only fastq pass reads were used for further processing and genome assembly. The reads were first filtered based on quality using the Filtlong package v0.2.0 (https://github.com/rrwick/Filtlong), and then adaptors were removed using the Porechop package v0.2.4 (https://github.com/rrwick/Porechop. Minimap2 v10.2.0 [72] was used to extract mitochondrial reads through the sequence alignment of processed reads to the published *M. pinodes* mitochondrial genome [73]. The post-processing of sequence alignment data, including conversion from SAM to BAM format and the sorting of BAM files, before separating mapped and unmapped reads as fastq files, was carried out using Samtools v1.12 [74]. Mapped reads were processed to assemble mitochondrial genomes, and unmapped reads were processed to generate nuclear genome assemblies for each isolate. Nuclear genomes were assembled with CANU v2.0 [75]. Approximate genome sizes for all three species were determined using CANU, wtdgb2 v2.2, [76] and Flye v2.6 [77] for input into CANU: *P. pinodes* (35 Mbp), *P. pinodella* (45 Mbp), *P. koolunga* (60 Mbp). Assemblies were carried out with three different minReadLength values (1000, 3000, 5000), and the assemblies with the lowest number of contigs were chosen as the final assemblies for each isolate. These draft assemblies were polished three times using Racon v1.4.10 [78] and Pilon v1.23 [79]. The nuclear assemblies for Isolate1Pk, Isolate2Pk, Isolate3Pp, Isolate4Pp, Isolate5Pp had Illumina short-reads available on the SRA database (SRR8284140, SRR8284141, SRR8284142, SRR8284136, SRR8284138, SRR8284139), and these were used for the hybrid Pilon polishing. The assembled genomes were assessed for quality using Quast v5.0.2 [80], and scaffolding was carried out using LRScaf v1.1.10 [81] and MeDuSa v1.6 [82]. The completeness of the genome assemblies was determined using BUSCO [83], and duplicated genes were removed using PurgeDup v20201029 [84]. Final genome assemblies were again assessed using the BUSCO Fungi_Odb10 database on GenSAS (JL Humann, et al., 2019).

Known fungal single-copy genes, elongation factor 1-α (EF1-α), elongation factor 3-α (EF3-α), and the second largest subunit of RNA polymerase II (RPB2) were used to test for duplications in the assemblies that would indicate assembly errors using Blastn. The sequences used included *Aspergillus tanneri* translational elongation factor EF-1α (TEF3; XM_033570262.1), *Ascochyta pisi* strain CBS 122785 DNA-directed RNA polymerase II second-largest subunit (RPB2; MT018244.1), and *Aspergillus oryzae* strain translation elongation factor 1- α (tef-1-α; JCM10114).

The mitochondrial genomes were assembled using wtdgb2. The assemblies were quality checked by increasing the value of the estimated genome size parameter -g (65, 70, 80) in order to confirm the mitochondrial genome size and contig number before polishing three times using Racon version 1.4.10.

### 2.7. Genome Annotation

Automated genome annotation was carried out using the GenSAS web server platform, which includes pipelines and databases developed for fungi accessed on 11 Jan 2021. The transcriptome data generated above were used for the further refinement of the gene annotation. Detailed annotation of repeats in the genome assemblies was carried out using RepeatMasker v4.0.7 [85] and RepeatModeler v1.0.11 (http://www.repeatmasker.Org. RIPper [86], a web-based tool, was used to identify repeat-induced mutation activity in the assembled genomes of the reference isolates for each of the three AB species. Repeats were masked for all of the assemblies before the functional and structural annotation was performed. Transcripts were predicted and aligned using BLAST nucleotide (blastn) [87], BLAT [88], and Program to Assemble Spliced Alignments (PASA) [89] against NCBI refseq fungi databases with default parameters. Trembl, SwissProt (https://www.uniprot.org/) and DIAMOND proteins v0.9.22 [90] were used to predict the protein sequence with NCBI refseq fungi and database default parameters. Gene prediction was performed using Augustus v3.3.1 [91] with the *A. fumigatus* library as a hint annotation model, GenemarkES v4.38 [92], and Braker v2.1.1 (https://github.com/Gaius-Augustus/BRAKER). The most complete prediction was used for further downstream annotation. RNAmmer tool v1.2 [93] was used to annotate 5s/8s, 16s/18s, and 23s/28s ribosomal RNA in the nuclear genomic sequences. SSR Finder v1.0 (http://www.fresnostate.edu/ssrfinder/) was used to find Simple Sequence Repeats (SSR), and tRNAscan-SE v2.0 [94] was used to identify tRNA genes in the AB genome assemblies. Functional annotation was carried out to enable the assignment of function and gene names. Conserved protein domains for the gene models were annotated using BLAST protein vs protein (blastp) version 2.7.1, DIAMOND, InterProScan v5.29–68.0 [95], and PFAM v1.6 [96]. SignalP v4.1 [97] was used to predict the presence and location of signal peptide cleavage sites in the predicted protein sequences. The completeness and quality of the structural annotation was assessed using Benchmarking Universal Single-Copy Orthologs (BUSCO) v3 with the Fungi_Odb10 database. GC content analysis was carried out using a 1000k sliding window.

Mitochondrial genomes were annotated using web servers for MITOS2 (http://mitos.bioinf.uni-leipzig.de/index.py accessed on 28 May 2021) [98], RNAweasel, and MFannot (https://megasun.bch.umontreal.ca/RNAweasel/; accessed on 28 May 2021). Codon usage was calculated using the web server, a Sequence Manipulation Suite (http://www.bioinformatics.org/sms2/codon_usage.html; accessed on 28 May 2021).

### 2.8. AB GC Content Variation

Each genome was cut into non-overlapping 1 kb pieces, and the GC content of each 1 kb piece was measured. The %GC for each piece was rounded to an integer value and summed by the %GC value for each genome to create a %GC frequency distribution.

### 2.9. AB Nuclear Genome Structural Variation and Synteny Analysis

Genome-wide comparisons of structural variation between pairs of AB reference genome assemblies were carried out using Nucmer v4.0 [99] to generate delta alignment files that were used as input into Assemblytics [100]. Genome similarity data were generated for reference isolates (Isolate2Pk *P. koolunga*, Isolate3Pp *P. pinodes* and Isolate18Ppll *P. pinodella*) using Nucmer with the --mum setting. The identification and analysis of the genome similarities among AB isolates was carried out using CIRCOS software v 0.69–9 [101] using the links function to illustrate genomic regions identified by Nucmer to have a high level of nucleotide sequence homology.

### 2.10. BLASTn Similarity and Blast2go Analysis

Sequence similarity checks for the assembled nuclear genomes against the NCBI fungal genome nr/nt database were carried out using blastn, blastp and blastx. The parameters were set to allow only the top hits for each contig in the assembly being queried. Blast2go software was used to generate the top blast hits for the genome assemblies using annotated CDS gene files for reference isolates within each AB species.

### 2.11. Mating Type Determination

*A. lentis* MAT1-1 (DQ341314.1) and MAT1-2 (DQ341315.2), as well as *P. pinodella* MAT1-1 (JF815529.1) and MAT1-2 (JF815531.1) genes (accessed on 25 February 2021), were blasted against assembled nuclear genomes using blastn to determine their mating type according to homologous genes described by Cherif et al. (2006).

### 2.12. Ortholog Analysis

Predicted protein sequences in FASTA format for the AB species were used to predict orthologous gene clusters with the web program OrthoVenn [102], which utilizes OrthoMCL, a well-known heuristic approach for the identification of ortholog groups [103]. Gene families were analysed, and core and accessory genes were identified and used to describe pangenomes for the respective field pea AB species.

### 2.13. Phylogenetic Analysis

Whole-genome phylogeny for the field pea AB isolates was determined using kSNP3 [104]. The resulting parsimony tree was uploaded into FigTree version 1.4.3 (http://tree.bio.ed.ac.uk/software/figtree/) for drawing, rooting, and ordering nodes. OrthoFinder version 2.3.8 [105] was also used to infer phylogeny using predicted protein sequences. Fourteen fungal species sourced on 4 February 2021 from public databases (NCBI) were included in the analysis, in order to determine the phylogenetic relationships. All of the species used in the analysis are from the phylum Ascomycota, except *Ustilago maydis,* which is from the phylum Basidiomycota. Using Nei’s pairwise genetic distance calculation method, neighbour-joining (NJ) dendrograms were generated for rDNA internal transcribed spacer (ITS) and protein coding genes β-tubulin, the second largest subunit of RNA polymerase II (RPB2), transcription elongation factor 1-α (TEF1α) and topoisomerase 1 (TOP1), with NCBI accession numbers GU237883.1, KX838493.1, AB007770.1, and NW_017306212.1, respectively (accessed on 11 February 2021).

### 2.14. Carbohydrate-Active Enzyme (CAZymes) Analysis

CAZymes annotation was carried out using the dbCAN2 annotation pipeline (http://bcb.unl.edu/dbCAN2/, accessed on 26 August 2021), a web-based application [106]. This application annotates for putative homology to protein sequences in the CAZymes database using three tools: (i) HMMER, a dbCAN HMM (hidden Markov model) database [107]; (ii) DIAMOND, a CAZy pre-annotated CAZyme sequence database [90]; and (iii) Hotpep, a conserved CAZyme short peptide database [108].

### 2.15. Secondary Metabolite Gene Cluster Analysis

Secondary metabolite biosynthetic clusters were identified using the antiSMASH web server (fungal version) (https://fungismash.secondarymetabolites.org/, accessed on 12 February 2021) with the default settings [109,110].

## 3. Results

### 3.1. Genome Sequencing

The genome sequencing of 18 AB isolates (Supplementary Table S1), based on Oxford Nanopore long-read sequencing technology, produced draft genome assemblies that were near complete. The raw sequencing data size generated for the isolates ranged from 2.9 Gb to 30 Gb, the total sequencing read length ranged from 2 Gbp to 15 Gb, and the sequencing coverage ranged from 40× to 339× (Supplementary Table S2). After adaptor removal and quality filtering, the total read length was between approximately 2 Gb and 9 Gb, and coverage was from 32× to 197×. Isolate 32 had the lowest total read length, and isolate18 had the highest. The number of raw RNA reads generated from different isolates ranged from 6.9 M to 50 M, with the lowest being for isolate42 and the highest being for isolate1 (Supplementary Table S3). More than 90% of the total reads had a quality score of more than 20 (Q20), and the average GC content was approximately 55%.

### 3.2. Genome Assembly Statistics

Some of the genome assemblies were assembled at a near-chromosome-level resolution. The number of scaffold/pseudochromosomes ranged from 12 to 270, with an average genome length of 56.7 Mbp, 37.5 Mbp and 35.1 Mbp for *P. koolunga*, *P. pinodella* and *P. pinodes,* respectively (Table 1). The scaffold N50 averages were 2.9 Mbp; 2.9 Mbp and 3.0 Mbp, and the average GC% content was 44%, 51% and 52% for *P. koolunga*, *P. pinodella* and *P. pinodes*, respectively. An assessment of the completeness of the assemblies, determined using Quast v5.0.2, indicated that all of the isolate genome assemblies were greater than 93% complete, with the highest percentage of 99% being attained for assemblies that were polished with Illumina reads. Isolate2Pk, Isolate3Pp and Isolate18Ppll were designated as species reference genomes based on their high BUSCO completeness and low scaffold number.

### 3.3. Genome Annotation

A higher number of genes were predicted using Augustus compared to Braker and GenemarkES, with the latter two programs being similar in their predicted number of genes (Table 2). BUSCO assessment of annotation, using the Fungi 10db dataset, showed that GenemarkES resulted in the highest completeness percentage for most isolates, and Augustus was the lowest. Annotation data from GenemarkES, with a predicted number of genes ranging from about 9700 to 12,900, was used for all of the subsequent comparative analysis. tRNAs and rRNAs annotated using tRNAscan-SE and RNAmmer on GenSAS, respectively (Table 2), indicated that the average numbers of tRNAs among the isolates were 138, 139, and 129, and those of rRNAs were 82, 69 and 73 for *P. koolunga*, *P. pinodella* and *P. pinodes*, respectively. We observed that the total rRNA number for most isolates was under 100; however, three isolates (one in each AB species) had over 100 rRNAs.

### 3.4. Blastn and Blast2go Analysis

Nucleotide BLAST analysis of the predicted genes for AB isolates against the NCBI nr/nt databases revealed sequence similarity to other species in the Ascomycota phylum. High-scoring matches for the scaffolds included *Parastagonospora nodorum, Alternaria* sp., *Leptosphaeria* sp., *Didymella rabiei, Ascochyta pisi, Phoma* sp. *1 OB-2014,* and *Bipolaris* sp. for the nt database, and *Ascochyta rabiei* for nr database. Blast2go analysis showed *A. lentis* and *A. rabiei* as top blast hits for *P. koolunga. Didymella keratinophila* and *Didymella heteroderae* were top hits for *P. pinodes* and *P. pinodella* (Supplementary Figure S1). *D. keratinophila* and *D. heteroderae* are human fungal pathogens, and emerge closest to the AB species, likely because they are *Didymella* species similar to *P. pinodes* (previously *Didymella pinodes*) and *P. pinodella.*

### 3.5. AB Nuclear Genome Structural Variation (SVs) and Synteny Analysis

**Figure 2:**
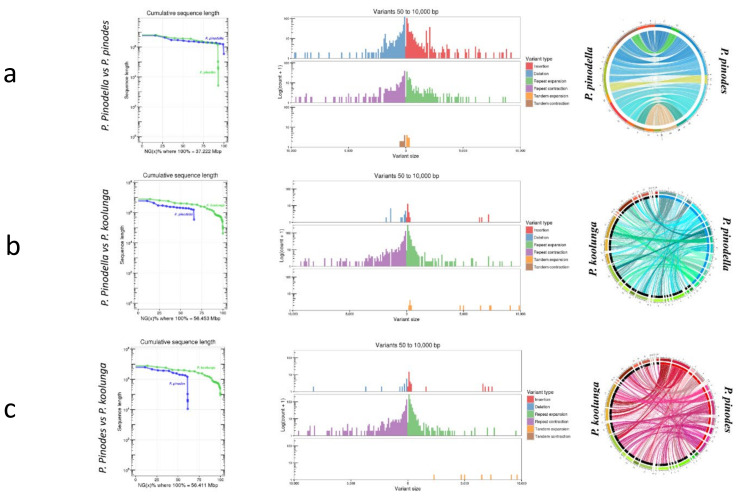
Structural variant (SV) analysis and Circos plots of AB species’ nuclear genomes between reference Isolate3Pp (*P. pinodes*), Isolate18Ppll (*P. pinodella*) and Isolate2Pk (*P. koolunga*). (**a**) SV analysis showing the higher occurrence of indels compared to repeats between the species and syntenic homology of *P. pinodes* (16 scaffolds, red) and *P. pinodella* (16 scaffolds, blue). (**b**) A comparison of *P. pinodella* (16 scaffolds, blue) and *P. koolunga* (29 scaffolds, black) reveals that repeat expansion and contraction are the most prevalent SV. Less homology is observed in (**b**) compared to (**a**). (**c**) Similarly to (**b**), a comparison of *P. pinodes* (16 scaffolds, red) and *P. koolunga* (29 scaffolds, black) shows that the most prevalent SV is repeat expansion and contraction, and there is less homology between the species compared to (**a**).

Differences in gene sequence and presence–absence variation play a key role in the specificity for host and cultivar in closely related plant pathogenic fungi. These variations have been linked to the conferring of advantageous qualities to the isolate. A whole-genome comparison of AB species using the assembled genomes of reference isolates for each of the three species revealed structural variants between AB species, including insertions, deletions, tandem expansions/contractions, and repeat expansions/contractions (Figure 2). The alignment of *P. pinodes* and *P. pinodella* showed that insertions and deletions were more prevalent, while the alignments of *P. koolunga* with either *P. pinodes* or *P. pinodella* showed that repeat expansion and contraction were the most prevalent SVs. The SVs observed between the AB species were mostly between 50 and 2500 bp. The total number of structural variants was higher for *P. pinodella* vs. *P. koolunga* (1176) compared to *P. pinodella* vs. *P. pinodes* (1155) and *P. pinodes* vs *P. koolunga* (1146). The total bases affected by structural variants on the other hand was greater for *P. pinodella* vs. *P. pinodes* (1.27 Mb) compared to *P. pinodella* vs. *P. koolunga* (0.87 Mb) and *P. pinodes* vs. *P. koolunga* (0.87 Mb). Nucmer considers only the regions of the genomes that align to each other, and so the SVs observed between the species only depict regions that are homologous between the species. Dot plots of alignment showed overall good collinearity between *P. pinodella* and *P. pinodes,* as well as indels, inversions and translocations (Supplementary Figure S2). A marked difference in genome rearrangement was observed between *P. koolunga* and the other two AB species. The nuclear genomes of the AB isolates plotted using Circos revealed genome similarity and conservation. The colored rings in the plot link annotate genes which were found to be conserved between the species, while the white spaces show absence of homology (Figure 2). Gene conservation was observed among the AB species, with a higher degree of conservation and synteny between *P. pinodes* and *P. pinodella* compared to *P. koolunga*.

### 3.6. Repeat Analysis of Genome Assemblies

Fungal genomes have highly variable sizes and compositions, which contribute to genome plasticity and expansion, which is typically driven by the activity of repeat elements [111]. Repeat analysis using RepeatMasker and RepeatModeler indicated some degree of overlap, although many more repeats were observed from RepeatModeler analysis. Elements such as Copia, Gypsy, Mariner, Tad1 and simple repeats were annotated by both RepeatMasker and RepeatModeler. *P. koolunga* genomes were found to have a greater quantity of repeat elements compared to *P. pinodes* and *P. pinodella* (Supplementary Figure S3). RepeatModeler showed that, on average, 44% of the *P. koolunga* genome sequence contained repeat elements, whilst RepeatMasker only indicated 11%. Simple repeats were the most abundant, followed by Gypsy (Supplementary Table S4). Unique repeat elements that were observed with RepeatModeler but not with RepeatMasker included hAT-Restless, TcMar-Tc1, TcMar-Fot1, L1-Tx1 and DNA/Academ. L1 and DNA/Academ were only present in *P. pinodella* isolate113 and isolate18, respectively. BCBOTYPOL and POT2 were only observed in *P. koolunga* and *P. pinodella*, respectively. Isolate-specific repeat elements FTF1 and AFLAV were observed for *P. pinodella* Isolate104Ppll and *P. koolunga* Isolate22Pk, respectively.

**Figure 3:**
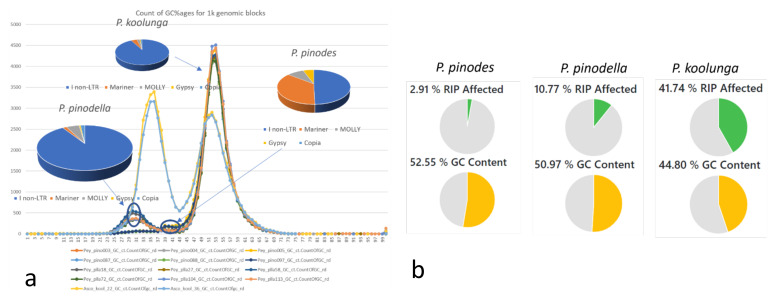
Repeat analysis. (**a**) GC content variation among AB species, with the top five TE expressed as a pie chart for each AB species. (**b**) Total RIP-affected regions per genome assembly for the AB species using the reference isolates for comparison.

GC content variation analysis (Figure 3a) showed a distinctive bimodal distribution for *P. pinodella* and *P. koolunga,* with *P. koolunga* having near-similar bimodal peaks. *P. pinodes* shows a strong primary peak with what seems to be a very slight secondary peak much closer to the primary peak compared to those observed for *P. pinodella* and *P. koolunga*. RIP (repeat-induced point mutation) analysis using the web browser tool RiPPER revealed large RIP-affected genomic regions. The results showed a significant impact to a total of 0.5 Mb, 3.7 Mb and 22 Mb of the genomes of *P. pinodes*, *P. pinodella* and *P. koolunga*, respectively. *P. koolunga* shows the most significant impact by RIP activity, followed by *P. pinodella*, which is proportional in pattern to the RIP percentage (Figure 3b) observed and the repeat analysis results acquired for the AB species.

### 3.7. Repeat Analysis using Transcriptome Data

In order to investigate whether the repeats annotated in the genomes are active, we carried out transcriptome analysis of the repeat elements observed in the genome. The results (Table 3) showed that I_1_A0, a non-LTR retrotransposon of the i clade, is the most expressed repeat of the AB species, although this result was split among the *P. pinodes* isolates. Three *P. pinodes* isolates (Isolate 4Pp, Isolate5Pp and Isolate87Pp) showed that Mariner, a DNA transposon, was the most expressed transposable element (TE); it comprises mobile DNA sequences that move within the genome. *P. pinodes* (53,361) and *P. pinodella* (68,806) had much higher read counts of repeat expressed compared to *P. koolunga* (8062), which has the highest repeat content in its genome (Table 3).

Observing the top five most expressed TE in the AB species showed that *P. pinodella* and *P. koolunga* have a similar repeat expression profile compared to *P. pinodes* (Figure 2a), despite being more phylogenetically distant. Mariner and Gypsy are expressed at higher levels in *P. pinodes* compared to the other AB species, and Copia is expressed at higher levels in *P. pinodella*. There are also some species-specific TE expression patterns which were observed, such as AFUT1 specific to *P. koolunga*, CFT1 and TFO1 specifically expressed in *P. pinodes,* and POT2, which was only observed to be expressed in *P. pinodella* (Table 3).

**Figure 4:**
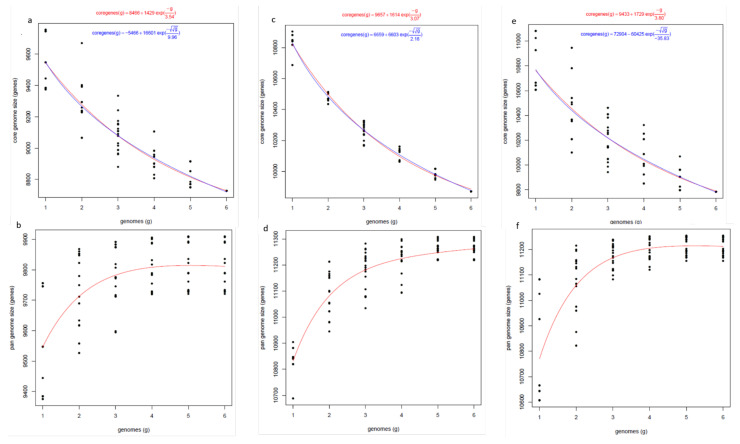
Pangenome analysis of the cDNA sequence (CDS) annotation for AB species. Core and pan-genome growth simulation plots for the AB species. Isolate CDS data were added in a random order by adding up six permutation experiments per species. *P. koolunga* (Panel (**a**,**b**)), *P. pinodella* (Panel (**c**,**d**)), and *P. pinodes* (Panel (**e**,**f**)).

For the pangenome analysis, we determined the number of core and accessory (isolate specific) genes using Get-HomologueEST. The number of genes shared within species, upon the sequential addition of new isolates from the same species, was extrapolated by the program. The results of all of the permutations of our six isolates for each species are shown in Figure 4. In order to remove low-confidence sequences, only the clusters found in at least two genomes were included in the pangenome analysis. For all of the AB species, the number of core (shared) genes decreased with successive additions of new genome sequences. The pangenome size for our AB species, as expected, increased with the number of genomes added, and began to plateau at six genomes, which indicated the possibility of a closed genome; however, the addition of more genomes would be required to confirm this.

### 3.8. Mating Type Determination

The mating type gene analyses for *P. pinodes* and *P. pinodella* were consistent with the literature, with *P. pinodes* being homothallic and *P. pinodella* being heterothallic (Supplementary Table S5). While *P. pinodella* MAT genes did not identify any mating type genes in *P. koolunga*, *A. lentis* MAT gene analysis identified *P. koolunga* as being heterothallic.

### 3.9. Orthogroup Analysis

Orthologous cluster analysis carried out using Orthovenn, as summarised in Table 4, indicated a higher number of protein clusters in *P. pinodella* and *P. pinodes* isolates (11,782; 11,653) compared to *P. koolunga* isolates (10,305). A substantial number of orthologous clusters were observed in *P. pinodella* (3543) compared to the other AB species, whereas for single-copy orthogroup clusters *P. pinodes* (9215) was observed to be greater compared to the other species.

**Figure 5:**
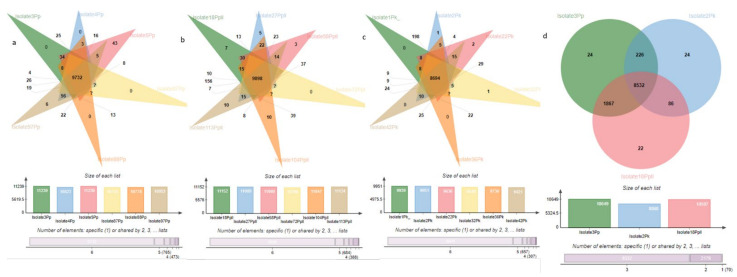
Venn diagrams of AB inter and intra-species orthology showing shared gene families (gene clusters). Venn diagrams and summary tables showing orthologs for the reference isolates *P. pinodes*, *P. pinodella*, and *P. koolunga.* (**a**) Orthologous gene clusters among *P. pinodes* species. (**b**) Orthologous gene clusters among *P. pinodella* species. (**c**) Orthologous gene clusters among *P. koolunga* species. (**d**) Orthologous gene clusters between the three AB species using the reference isolates Isolate2Pk, Isolate3Pp, and Isolate18Ppll.

The analysis of the orthologous clusters shared by all of the isolates within a species revealed the extent of the homology within that species. A greater homology in *P. pinodella* (9898) was observed (Figure 5a–c), and *P. koolunga* (8694) had the least observed homology among species. *P. pinodes* Isolate5Pp (43) had the most unique orthologous clusters.

The comparison of the orthologs against the AB reference isolates resolved 10,781 protein clusters, of which 2412 were shared by at least two species and 8532 were common among all of the species (Figure 5d). There were some unique clusters observed for all of the AB species. Gene ontology (GO) annotation of the unique clusters revealed proteins that are unique to each AB species (Table 5). The number of clusters shared between *P. pinodes* and *P. pinodella* was higher (1867) than that between *P. pinodes* and *P. koolunga* (226), and *P. pinodella* and *P. koolunga* (86).

### 3.10. Phylogeny

A phylogenetic tree using the whole-genome assemblies for 18 AB isolates was generated. The tree was rooted using *Ustilago maydis* as an outgroup, and the nodes were ordered. The clustering showed the clear separation of the different AB species (Supplementary Figure S4a). OrthoFinder was used to resolve phylogeny using Protein fasta files from the respective genome annotations. Analysis with 13 ascomycete species, including *A_fumigatus*, *A_lentis*, *L_maculans*, *C_albicans*, *A_alternata*, *A_brassicicola*, *A_tenuissima*, *A_rabiei*, *A_arborescens*, *S_cerevisiae*, *N_crassa*, *F_oxysporum* and *U_maydis,* as well as the field pea AB species, identified 341,319 total proteins (95.3% of total) assigned to 17,621 orthologous gene clusters. The OrthoFinder results showed that 50% of all of the proteins were in orthogroups containing 29 or more proteins (G50 = 29), and were contained in the largest orthogroups (O50 = 4714). There were 2221 orthogroups with all of the species present, and 547 of these consisted entirely of single-copy orthogroup proteins. A phylogenetic tree was generated using 1189 orthogroups with minimum of 93.3% of species having single-copy proteins in any orthogroup. The ortholog phylogeny of the AB species (Supplementary Figure S4b) showed that *P. pinodes*, *P. pinodella*, *P. koolunga*, *A. rabiei* and *A. lentis* diverged from a common ancestor. Evolutionary events led to the divergence in the tree, with *P. pinodes* and *P. pinodella* being more closely related to each other, and *P. koolunga* being more closely related to *A. rabiei* and *A. lentis*.

The percentages of core (84%) versus variable (16%) genes from Orthovenn2 were highly similar across the species, although the number of genes in each class was different (Supplementary Table S6). The Orthovenn2 results were confirmed using OrthoFinder, and the results were very similar, with only small variations.

### 3.11. Gene Duplication Events

OrthoFinder considers gene events to be well supported when both copies of the duplicated gene are retained in at least 50% of the descendant species. Seventy-eight well-supported gene duplication events were revealed at node N8 (Supplementary Figure S4c), leading to the speciation of *P. pinodes, P. pinodella, P. koolunga, A. rabiei* and *A. lentis*. One hundred gene duplication events occurred at node N11, where *P. pinodes* separated from *P. pinodella*, and further duplication events took place to differentiate the isolates within these species. Furthermore, at node N10, 29 gene duplication events led to the speciation of *P. koolunga*, *A. rabiei* and *A. lentis*.

### 3.12. CAZyme Analysis

**Figure 6:**
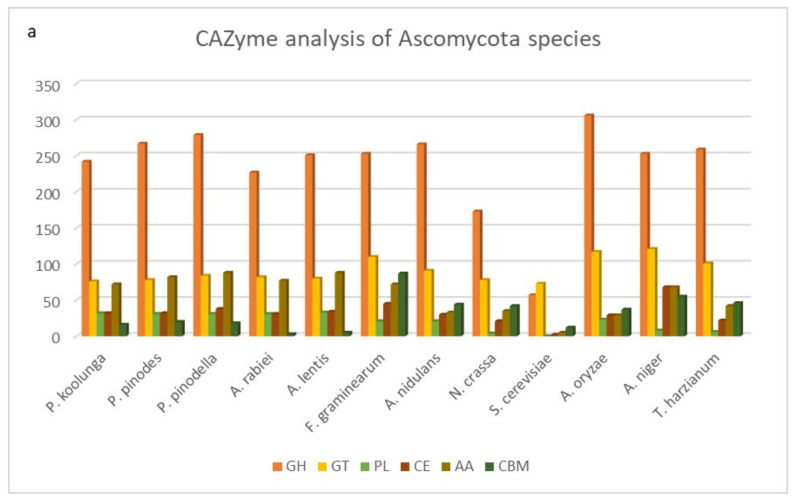

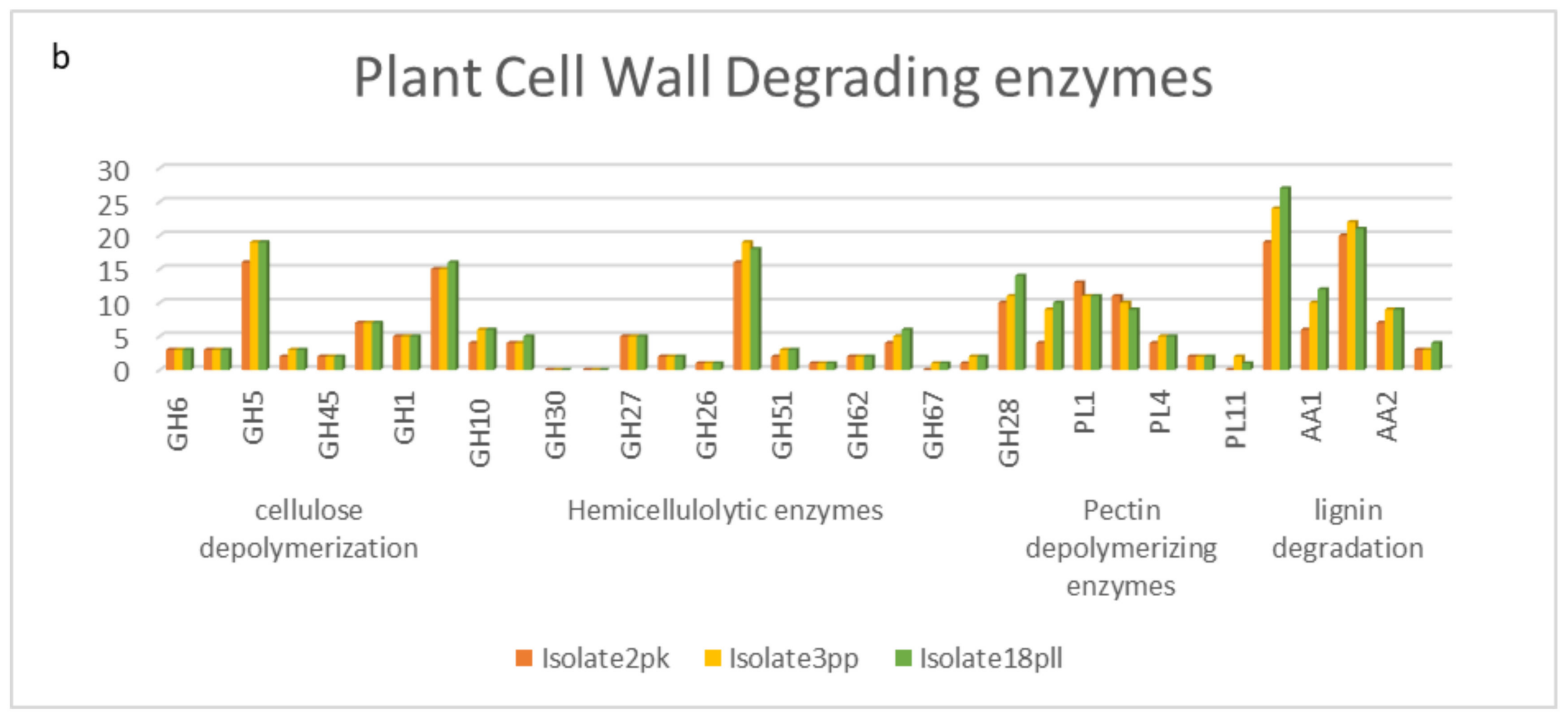
AB CAZyme analysis. (**a**) CAZyme analysis profile of Ascomycota species; (**b**) AB Plant-cell-wall-degrading CAZyme analysis (GH- glycoside hydrolases, GT- glycosyl transferases, PLs- polysaccharide lyases, CEs- carbohydrate esterases, AAs- auxiliary activities and CBMs- carbohydrate-binding modules).

The degradation of plant cell walls plays an important role in the colonization and pathogenicity of plant pathogenic fungi. The fungal CAZyme repertoire provides crucial information on their lifestyle. The annotation of carbohydrate active enzymes (CAZymes) performed using HMMER, DIAMOND, Hotpep and CGCFinder indicated that the annotated number of CAZymes ranges from 457 to 609, which was on average 4.6% of the total predicted proteins for these isolates (Supplementary Table S7). CAZymes were grouped according to their classification, which included glycoside hydrolases (GHs), glycosyl transferases (GTs), polysaccharide lyases (PLs), carbohydrate esterases (CEs), auxiliary activities (AAs), and carbohydrate-binding modules (CBMs), as shown in Supplementary Table S7. The results revealed that GHs were the most abundant CAZyme class for all of our AB isolates, followed by GTs and AAs, with the least abundant CAZyme class being CBMs. All of the AB isolates across the three species showed similar CAZyme profiles. There was a positive correlation observed between the total number of CAZymes and the number of predicted protein coding genes (PCGs) in the genome (Supplementary Figure S5a). The total number of CAZymes and the distribution of different CAZyme classes for reference Isolate2Pk (*P. koolunga*), Isolate3Pp (*P. pinodes*) and Isolate18Ppll (*P. pinodella*) were compared to other Ascomycota species (Figure 6a) and, collectively, similar profiles were observed with other published AB species, *Ascochyta rabiei* and *A. lentis*.

The analysis of plant-cell-wall-degrading CAZymes (Figure 6b) revealed an abundance of lignin degradation enzymes AA3 and AA9. The next most abundant CAZymes were β-glycosidases (GH3), cellulases (GH5) and the hemicellulolytic enzyme α-arabinosidase (GH43). The pectin depolymerizing enzymes included polygalacturonases (GH28 and GH78) and polygalacturonic lyases (PL1, and PL3). For the ascomycete-specific CAZymes GH94, GH130, PL10 and GH76, analysis showed from seven to 11 copies of GH76, one to two copies of GH94, and no GH130 or PL10, in our dataset. For CAZymes involved in fungal cell wall degradation, the analysis revealed the highest numbers for chitinase (GH18) and glucanase (GH16) compared to the other CAZymes such as GH27, GH92, and GH79 (Supplementary Figure S6).

### 3.13. Secondary Metabolite Gene Cluster Analysis

The analysis of biosynthetic gene clusters (BGCs) using the fungal AntiSMASH web server identified 19 to 28 BGCs per genome (Supplementary Figure S7a). The BGCs observed included non-ribosomal peptide synthase (NRPS, NRPS-like), polyketide synthase (PKS), terpene, indole cluster, ribosomally synthesized and post-translationally modified peptides (RiPPs), β-lactone, and various hybrids of PKS/NRPS clusters. Fungal-RiPPs and β-lactone were specific to *P. pinodella* and *P. pinodes* species. The hybrid NRPS/T1PKS/indole was found in only isolate1Pk of *P. koolunga*. *P. koolunga* species generally had higher numbers of NRPS and T1PKS (Type1 PKS) clusters, while *P. pinodella* and *P. pinodes* generally had higher numbers of terpene clusters (Supplementary Figure S7b–d).

### 3.14. AB Species Mitochondrial Genomes

Sequencing reads that mapped to the *M. pinodes* mitogenome were assembled using wtdgb2, which resulted in single mitochondrial contigs for all of the AB isolates. The published *M. pinodes* mitogenome (55.9 Mbp) is very similar in length to the *P. pinodes* mitogenome assembled in this study (Table 6). The total base pair length ranged from 48 kb to 73 kb, with *P. koolunga* isolates having the largest mitochondrial genomes and *P. pinodella* isolates having the smallest. The GC content was between 28% and 29% for all of the isolates (Table 6). The genes identified included twelve core protein coding genes and two ribosomal RNA coding sequences and tRNAs corresponding to all 20 amino acids, with total numbers ranging from 25 to 29 (Supplementary Table S8). The NADH-dehydrogenase subunit 5 encoding gene *(nad5*) copy number was significantly lower in *P. koolunga* (3–5) compared to the other AB species (8–13). In addition, two putative mitochondrial ribosomal protein genes (*rps3* and *rps5*) were identified for the AB isolates, as well as two mitochondrial ribosomal RNA genes (*rrnL* and *rrnS*). The DNA-dependent RNA polymerase gene (*rpo*) was only observed in *P. koolunga* isolates. A graphical representation of the genomes for the reference isolates was drawn using CHLOROBOX (Figure 6a–c). The genes on the outside of the ring are from the direct strand, and the genes on the inside are from the reverse strand. RNAweasel analysis (Supplementary Table S9) revealed the presence of the RNA subunit of the mitochondrial RNAse P gene (*rnpB*). *P. koolunga* mitochondrial genes had the largest number of introns compared to the other AB species, followed by *P. pinodes*. *P. koolunga* species also had the highest number of mitochondrial tRNA genes compared to *P. pinodella* and *P. pinodes*.

The mitochondrial ribosomal protein gene *rps3* was observed to be present in all of the AB isolates; however, the *rps5* gene was observed only in some isolates across species, including Isolate1Pk, Isolate22Pk, Isolate42Pk, Isolate113Ppll, Isolate4Pp, and Isolate5Pp (Supplementary Table S8). The location of the *rps3* and *rps5* genes varied among the species. In *P. koolunga*, *rps3* and *rps5* genes were usually located between *nad1* and *nad2* genes; however, *rps3* was sometimes found between *trnL1* and *trnF* genes. In both *P. pinodella* and *P. pinodes*, *rps3* was generally located between *nad4* and *cob*.

Mitochondrial genome annotation revealed the presence of only Group 1 introns in all of the field pea AB isolates sequenced in the study. The total number of mitochondrial introns ranged from 34 to 47 (Supplementary Table S9), with *P. koolunga* having the highest number of introns in its mitochondrial genome and *P. pinodella* having the lowest number. The *Cox1* gene that encodes cytochrome c oxidase I had the highest number of introns in all of the AB species, followed by *cob* in *P. koolunga* and *P. pinodes* (Supplementary Table S10). In *P. pinodella*, *Cob*, *Cox2* and *Cox3* had the second-highest number of introns. Several homing endonucleases (HEs) from the family LAGLIDADG and GIY, which aid in the self-splicing of introns, were observed in all of the AB isolates. Potential trans-splicing was also detected for the *Cox1*, *Cox2*, *Nad5*, *Cob* and *Nad2* genes.

**Figure 7:**
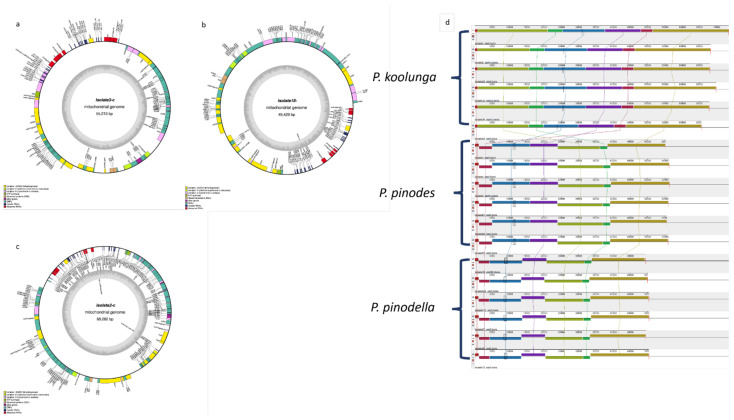
Graphical representation of the AB reference isolate mitochondrial genomes using OGDRAW from the CHLOROBOX web server. (**a**) *P. koolunga*, Isolate2Pk; (**b**) *P. pinodella*, Isolate18Ppll; and (**c**) *P. pinodes*, Isolate3Pp. (**d**) Mitochondrial gene order synteny among AB species.

Synteny analysis using Mauve showed mitochondrial gene order conservation across AB species (Figure 7). Highly syntenic homologous regions were detected within species, and variations in gene order and length were observed between species. *P. koolunga* species showed mitochondrial genome architectures that were distinct from *P. pinodes* and *P. pinodella*. *P. pinodes* and *P. pinodella* were quite similar, although an inversion was observed within *P. pinodella* species, as illustrated by the blue colinear block (Figure 7d).

Codon usage bias has been studied in order to understand the codon usage pattern of the AB species and has been shown in previous studies to provide insight into their evolutionary trajectories. Codon usage was observed to be similar in pattern among the AB species (Supplementary Table S11). Overall, *P. koolunga* had a slightly greater number of total amino acids, followed by *P. pinodes*. The codons with a significantly high frequency (>900) include Phe (TTT), Lys (AAA), Leu (TTA), Tyr (TAT), Asn (AAT) and Ile (ATA, ATT). There is about a two-fold preference of stop codon TAA usage compared to TAG across the AB species.

## 4. Diagnostics

Previous studies have shown that differentiating the AB species, particularly *P. pinodes* and *P. pinodella*, is challenging using traditional morphological methods. We mapped the field pea AB whole-genome assemblies to the most relevant fungal barcoding genes, which included protein coding genes, which are known for resolving fungal species. The comparison of AB genome assemblies to fungal barcoding genes such as internal transcribed spacer (ITS), RPB2, transcription elongation factor 1α (Tef1), β-tubulin and topoisomerase 1 (TOP1) was consistent with the whole-genome phylogeny in revealing that *P. koolunga* is more distantly related to the other AB species. It was difficult to distinguish *P. pinodes* from *P. pinodella* using ITS, β-tubulin and RPB2. Tef1 and TOP1 were found to be the best candidates for species differentiation (Supplementary Figure S8a–e) between the two *Peyronellaea* species. Furthermore, genetic distance based on kmers was used to build a parsimony mitochondrial phylogenetic tree (Supplementary Figure S8f) showing the mitogenome relationship among the AB species. The clear separation of *P. pinodes*, *P. koolunga* and *P. pinodella* was observed, as is consistent with the phylogeny determined using whole-genome assemblies.

## 5. Discussion

Although AB of field peas has been studied for many years, with literature on the subject dating as far back as 1944 [112], there remain significant gaps in the literature that limit our understanding of the essential features of the disease. Elements such as the molecular basis underlying the pathogenesis, pathogen–complex interaction, host–pathogen interaction, and disease epidemiology require the detailed genome analysis of the pathogens causing AB. This study reports the first whole-genome assemblies and comparative analysis of *P*. *koolunga*, *P. pinodella* and *P. pinodes*, the three primary species of the AB complex in Australia. The draft genome assemblies generated from long-read sequencing were within the expected genome size for the Ascomycota phylum (average 36.9 Mb), with some isolates having genomes larger than the average genome size for plant pathogenic fungi of 39.4 Mb [113,114,115]. The genome sizes for *P. pinodella* and *P. pinodes* at approximately 36 Mb were comparable to the assembled draft genomes of other Ascochyta species affecting legumes such as *A. rabiei* (40 Mb) [116] and *A. lentis* (42 Mb) [117]. However, a larger genome of approximately 56 Mb was observed for *P. koolunga*, which is unsurprising, as large genomes have been confirmed for Dothideomycete species [118,119,120]. The large genome size for *P. koolunga* is almost entirely due to a high proportion of repeat elements, including repeat expansion and contraction, and this could be the driving force for genome evolution in the species, as has been observed for *Pyrenophora teres* that cause net blotch in barley, with genome sizes ranging from 41 Mb to 51 Mb for the two *Formae speciales* f.sp. *maclata* and f.sp *teres* [121].

### 5.1. Near-Chromosome-Level Genome Assembly

The chromosome number for fungal species has been observed to range from as few as three (Zolan, 1995) to 21 (haploid) [117,122], which is comparable to our reference genome assemblies. The manual observation of our assemblies revealed the presence of TTAGGG repeats that are characteristic of fungal telomeres at the ends of *P. pinodes* and *P. pinodella* scaffolds. Pulsed-field gel electrophoresis of *A. rabiei* isolates from 21 countries, close relatives to the field pea AB species, revealed a chromosome number between 12 and 16. Other techniques such as optical mapping have been used to improve genome assemblies [123]. Optical mapping can further be used to improve assemblies such as Isolate22Pk with over 200 contigs and a sequencing depth of 32×. However, Isolate3Pp, Isolate18Ppll, Isolate72Ppll, and Isolate87Ppll, with scaffold numbers between 12 and 18 having had deep long-read sequencing coverage up to 190× with correction, even without optical mapping, are near chromosome-level assemblies [116,122]. There is a complete lack of descriptions of 1n and 2n forms of the AB fungi in the literature; the original descriptions of the species [17] and large taxonomic papers [6] lack this information. Information about ploidy is important in order to carry out genetic engineering and design efficient gene manipulation tools for the AB species. Although AB species ploidy was not estimated in this study, the assemblies generated can be used to determine it using computing tools such as nQuire and the K-mer counting approach [124,125]. The number of coding gene sequences (9717–12,249) was also within the range observed for Ascomycota species such as *N. crassa* (9820), *A. lentis* (11,638), and *L. maculans* (12,469). The rRNA and tRNA numbers were comparable to what has been found in the literature for fungal species within the Ascomycota phylum [116,117,126,127,128,129]. Overall, these AB genome assemblies are of high quality, and will be a valuable tool to facilitate the characterization of field pea AB populations, identify evolutionary drivers, and develop diagnostic and monitoring tools that would enable the advancement of durable and targeted management strategies.

### 5.2. AB Nuclear Genome Structural Variation

The synteny analysis of our isolates revealed high genomic conservation between species, mostly between *P. pinodes* and *P. pinodella* compared to *P. koolunga*. Host and cultivar specificity in closely related plant pathogenic fungi can be influenced by chromosomal rearrangements and presence–absence variations in key pathogenicity genes.

Chromosomal rearrangements were observed between the AB species, including the sectional translocation of genomic regions among species, which has been observed in other fungi, including *A. rabiei* and *A. lentis* [130,131,132,133]. The high repeat expansion observed for *P. koolunga* compared with other field pea AB species confirms that the large genome size of *P. koolunga* is likely due to large-scale evolution through repeat element expansion, and this could have an impact on pathogenicity and lineage-specificity, as genes near TEs could be altered, giving them new functions [121,134]. The presence of species- and isolate-specific TEs among AB species sheds some light on the level of genome diversity possible within AB populations. Simple sequence repeats (SSRs) are the most abundant in the AB genomes, followed by long terminal repeat (LTR) elements such as Gypsy. The most abundant TEs in the literature are the LTR retrotransposons Gypsy and Copia, and DNA Transposons such as Mariner. The percentages of repeat elements and TEs in fungal genomes have been reported to vary significantly among species [120,135,136,137]. TEs are known to play diverse roles in the genome, including the promotion of genome variability, expansion, increased fitness, and adaptability [138]. The DNA transposon OPHIO was identified in the *P. pinodella* and *P. pinodes* genome assemblies. These have been reported to provide genomic responses to biotic and abiotic stressors in the environment, highlighting possible roles in these species [139]. Further investigation into these TEs will provide clarity on whether they are functional, and information about the specific roles they play in the AB species genome architecture.

The distinct bimodal distribution of GC content bias observed for *P. koolunga* and *P. pinodella* signifies the presence of large proportions of AT-rich regions, especially in *P. koolunga*, which is consistent with the BUSCO GC% obtained for the AB genome assemblies. Highly AT-rich genomes are usually associated with a high content of repeat elements such as transposable elements (TE) [36,140]. TEs have been linked to the conferring of advantages in genetic diversity, adaptation and environmental fitness when expressed in the genome [141]. This gave rise to the “two speed genome” model, which was developed to account for the fact that TEs were observed to be in gene-sparse areas of the genome, and were associated with effector genes [36,119,142]. However, the presence of a high percentage of repeat elements in the genome does not always translate to high expression rates, as we can see in the AB transcriptome repeat analysis results. *P. koolunga*, having the highest repeat percentage, seems to have the lowest number of expressed repeats compared to *P. pinodes* and *P. pinodella*. This could be due to the fungal mechanism designed against the propagation of transposable elements known as repeat-induced point mutation (RIP) [143,144]. RIP has been found to deplete GC content, thereby increasing AT content through irreversible transitions of cytosine (C) to thymine (T) [145]. In silico RIP analysis showed the presence of RIP in all of the field pea AB species analysed, with *P. koolunga* having the most extensive RIP (42%) compared to *P. pinodella* (11%) and *P. pinodes* (3%). This suggests that most of the repeats in the *P. koolunga* genome have been silenced, which explains why even though *P. koolunga* has the highest repeat content in its assembled genome, it expresses the lowest number of repeat elements in its transcriptome. RIP tends to occur during the sexual phase when dikaryotic cells proliferate [146]. Extensive RIP is often found in fungi with sexual reproduction capabilities [147], and given that the sexual stages of *P. pinodella* and *P. koolunga* have not yet been observed, this result suggests that *P. pinodella* and *P. koolunga* too might have the capacity for cryptic sexual reproduction, as has been reported for *Aspergillus* and *Penicillium* species [146], or might represent ancestral RIP activity, as has been observed in other asexual fungi [146,148,149]. Overall, the differences in the repeat content, both in the genome and transcriptome, suggest the AB pathogens have had different evolutionary trajectories, which should be taken into consideration when developing disease management strategies. The presence of diverse drivers of evolution between field pea AB species would elicit different host response genes among the species upon infection, as has been observed previously [150].

### 5.3. Orthologous Gene Cluster and Pangenome Analysis

Much more intra-species homology was observed among *P. pinodes* isolates compared to *P. pinodella* and *P. koolunga*. *P. pinodes* had more homology with *P. pinodella* compared to *P. koolunga*, corroborating our results from phylogeny. Pangenome analysis using protein homology did not show significant genome variation among AB species, with about 16% of the genomes accounting for accessory genes. However, some fungal species have been reported to have a higher percentage of accessory genes, such as the 63% reported for *Zymoseptoria tritici* [151]; the 16% observed in the AB species is sufficient to provide diversity in adaptability, virulence and pathogenicity in the field pea AB species, as has been observed for other fungal species, including *Saccharomyces cerevisiae* (~14%) and *Aspergillus fumigatus* (~16%) [46]. The gene ontology (GO) analysis of unique orthologous gene clusters revealed genes responsible for various metabolic processes and molecular functions, including pathogenesis. Interestingly, heterokaryon incompatibility protein 6 is unique to *P. pinodes.* This protein is responsible for non-self-recognition, which plays a crucial role in the vegetative recognition system in filamentous fungi. *P. pinodes* is the only species with a homothallic mating type, and so heterokaryon incompatibility protein 6 could play a role in the regulation of genotypic diversity and gene flow in the species.

### 5.4. AB Mating Type Determination

Mating type genes have been confirmed previously for *P. pinodella* and *P. pinodes* [152,153,154]. *P. pinodella* has been observed to exhibit a heterothallic mating pattern, having either one or the other mating type locus, and *P. pinodes* has been observed to be homothallic, having both mating types [154]. Our results from whole-genome sequencing agree with these findings, with heterothallism being evident for all of the *P. pinodella* isolates sequenced, and with homothallism being evident for all of the *P. pinodes* isolates. The two idiomorphs of the homothallic *P. pinodes* isolates were observed to be on the same scaffold and in close proximity, as has been reported for homothallic ascomycete species in *Gibberella zeae* [155] and *Chaetomium globosum* [156]. On the other hand, the mating type for *P. koolunga* has not been defined previously, and from our analysis we can conclude that the species exhibits a heterothallic mating pattern. This is similar to the mating type patterns observed for *A. rabiei*, *A. lentis* and *A. fabae*, which show a very close phylogenetic relationship to *P. koolunga*, as well as Phoma species such as *Phoma clematidina* [117,154,157,158,159].

### 5.5. Phylogeny

The congruence observed between the whole-genome and ortholog inference phylogeny of AB species showed a clear separation of the species, particularly *P. pinodella* and *P. pinodes*. The clear speciation of the AB species inferred in the phylogenetic analysis implies there is enough genetic variation between these species to be able to develop molecular markers for diagnostics. Previous research has reported the inability to differentiate *P. pinodella* and *P. pinodes* using *ITS*, and our results confirm this, showing *P. pinodella* and *P. pinodes* clustering together in the *ITS* dendrogram. Although protein coding genes have been proven to give a better resolution of the phylogeny, not all of them are able to provide a high resolution for all of the fungal species. Our results showed the low resolution of the species observed for *RPB2* and Beta tubulin. These AB species were classified using a CAPS assay based on *RPB2* gene, and the misidentification of some of the isolates has been observed, signifying the low resolution of the *RPB2* gene for AB species *P. pinodella* and *P. pinodes. Tef1* and *TOP1* displayed the high species resolution of *P. pinodella* and *P. pinodes*, making them strong candidates as barcoding markers for AB species identification.

Gene duplication events have been known to play important roles in the evolution of species by giving rise to new genes that code for proteins with potentially novel functions, which could include increased fitness and phenotypic diversity [160,161,162,163]. Here, we see that gene duplication played a major role in the speciation of the AB species separating *P. pinodes* and *P. pinodella*. Further analysis can be carried out to identify the specific genes duplicated, the resulting genomic structure, and the impact of the duplication events.

### 5.6. CAZyme Analysis

Predicted CAZymes also fall within the range of number and class distributions reported for plant necrotrophic fungi, with approximately 400–850 CAZymes in total, which is in line with the observed necrotrophic lifestyle for the field pea AB isolates [164]. The analysis of specific cell wall degrading enzymes reveals a high number of cellulose depolymerization, hemicellulolytic, pectin depolymerization and lignin degradation enzymes signifying major food sources for AB fungus. Pea has been reported to contain 32% hemicellulose, 27% cellulose, 41% pectin and at least 5.3% lignin. The AB species cell wall degradation repertoire reflects the enzymes that are responsible for successful pathogenicity of the pea plant [165,166]. An increased host range is equally proportional to the diversity of CAZymes in the fungal genome, as has been described for *Macrophomina phaseolina* (Islam MS, et al., 2012). The large CAZyme repertoire for the AB species suggests a broad host range, which is in congruence with host specificity experiments carried out for the species [167,168,169,170].

### 5.7. Secondary Metabolite Analysis

Fungal secondary metabolites (SMs) have been reported to play significant roles in pathogenicity, infection, and host specificity in necrotrophs [171,172,173]. Biosynthesis of fungal SMs is carried out by co-regulated genes, usually clustered at a genomic locus referred to as a biosynthetic gene cluster (BGCs). AB species’ genome-wide BCGs are in the range observed for *C. fulvum* (23) and *S. sclerotiorum* (29) which are also plant pathogenic fungi [174,175]. Ascomycete-specific PKS-NRPs hybrids were present in all of the AB isolates, and this agrees with their phylum identification [176]. Hybrid metabolites are known to be common in fungi, and we observed a few of them with moieties from different classes, including NRPS, T1PKS, and indole observed in one *P. koolunga* isolate.

Fungal-RiPPs are a class of BGCs that produce metabolites with a diverse variety of bioactivities involved in defence, competition, communication, and virulence [177,178,179,180]. RiPPs have been extensively characterized in plants and bacteria but are underexplored in fungi, although their first identification was in a fungus [181]. Fungal-RiPPs were observed in *P. pinodella* and *P. pinodes* species, indicating a possible role specific to these species compared to *P. koolunga*. Further characterization would be needed in order to identify the biosynthetic potential of these BCGs in these species. β-Lactones are also an underexplored class of fungal BGCs linked to several antimicrobial activities [182], and were also found to be specific to *P. pinodella* and *P. pinodes* species.

Terpenes and terpenoids play diverse roles in fungi: they can function as toxins, promote growth, and have antifungal properties [176,183]. The high production of terpenes has been observed in *Phoma sp*., which along with other volatile organic chemicals exhibit antifungal properties to certain fungal species, which is a possible defence mechanism [184]. However, it is unsurprising that *P. koolunga*, also a *Phoma sp*., exhibited a higher production of terpenes compared to the other AB species. Consequently, all of the AB species can be present on the plant at the same time, and so our results imply that the SMs repertoire of the individual AB species could either be tolerant to the other AB species in the complex or could serve to reduce competition; the latter has been reported [185].

Overall, the SMs diversity observed for the field pea AB species reveals that *P. pinodella* and *P. pinodes* species are more alike in their SM repertoire compared to *P. koolunga*. Although their host specificity overlaps, they seem to deploy different bioactive mechanisms, which could be why they elicit different host response genes, and resistance to one species does not translate to resistance to other species within the complex. *P. pinodes* is a broad host range fungus, being able to infect numerous legumes [169]; however, no research on host range specificity has been conducted for *P. pinodella.* Given the high genomic similarity between these two AB species, *P. pinodella* could possibly have a similar host range beyond field pea, similarly to *P. pinodes*.

### 5.8. Mitochondrial Genome Assembly

Although the mitochondrial genome size varied among the species and isolates, the numbers fall within the reported mitochondrial genomes for Ascomycota species [116,117,186,187,188,189]. There is a positive correlation between the mitochondrial genome length and number of introns. The genome size for *P. koolunga* was close to that reported for closely related species *A. lentis* and *A. rabiei*. The gene order observed shows high synteny within AB species. The number of protein coding genes observed is within that reported for several fungi [190,191]. Recent research characterizing introns and their HEGs across the fungi phyla reveal varying numbers of introns in protein coding genes, with the highest number in the *cox1* gene, followed by *cob* and *nad5* [190]. Genes with introns within AB species include *cox1*, *cox2*, *cox3*, *cob*, *nad1*, *nad5* and *nad4L*. Similar gene interruptions have been reported for *Aspergillus *sp., *Saccharomyces* sp., *Podospora* sp., *Neurospora* sp. and *Penicillium* sp. [188,190,192,193,194]. Interestingly, potential trans-splicing mediated by group 1 introns was observed within *cox1*, *cox2*, *nad5*, *cob* and *nad2* genes across all of the AB species. Trans-splicing has been reported in animals, plants, green algae, and fungi [195,196,197,198,199,200,201]. Most of these reports are for *cox1* genes, and nothing has been reported for *cox2*, *nad5*, *cob* and *nad2,* although Cis-spliced group 1 introns have been observed in *cox1* and *nad5* in fungi [202]. Additional analysis would be required in order to confirm this based on evidence of a ligated mature mRNA at exon–intron junctions and a complete intron RNA structure [195]. Furthermore, the presence of T7 phage-like single-subunit RNA polymerase (*rpo*) in only *P. koolunga* species suggests the insertion of a plasmid into its mitochondrial DNA. These *rpo* genes have been identified in other ascomycete fungi, and they are reported to be most likely non-functional, possibly fragmented, and eventually lost (Lang 2018). Previous research found that rpo was only found in species belonging to the fungal phyla Ascomycota, Basidiomycota and Chytridiomycota. Although rpo homologs were found in both *Ascomycota* and *Basidiomycota* fungal species, this was only in 10% of the Ascomycota species (227) sampled, compared to 57% for *Basidiomycota* (116) [203]. This means that *P. koolunga* is among a small percentage of species within the Ascomycota phylum that harbours an rpo homolog. Rpo-based phylogeny revealed both phyla-specific clades and rpo sequences that are shared between species of different phyla. This diversity of rpo sequences observed in the literature suggests a possible horizontal gene transfer between fungal species [203,204,205]. The mitochondrial codon usage for the AB species is similar to that reported for other ascomycete fungi [187]. A preference for AT-rich codons contributes to the low GC content (28–29%) in the AB species which has been observed for *Aspergillus* sp. and *Penicillium* sp. [188].

Phylogenetic analysis of the AB mitochondrial genomes reveals clear species separation, which can be further explored as barcoding markers for the species. This can be a standalone marker depending on its specificity to the species, or in addition to the other protein coding gene markers highlighted.

## 6. Conclusions

AB pathogens appear to have different evolutionary drivers, a broad host range as a result of their large CAZyme repertoire, a diverse secondary metabolite range, and mitochondria that are highly prone to mutation due to the action of homing endonuclease and repeat elements, which could have a significant impact on management strategies and the development of cultivar resistance. Plant pathogenic fungi make use of mitochondria proteins as well as nuclear proteins for the successful invasion, colonization, and pathogenicity of their plant host, provoking disease as well as encoding genes for resistance to host defense mechanisms. The 18 AB mt genomes developed in this study can enable future research into understanding AB mt biological significance with respect to disease pathogenicity, virulence, and function, even at the population level.

## Figures and Tables

**Table 1:** Genome assembly statistics of AB isolates. Isolates with double asterisks have illumina reads retrieved from NCBI; as such, hybrid assembly was carried out for them.

Isolates	No. of Contigs/SCAFFOLD	Largest Contig/Scaffold (Mb)	Total Length (Mb)	Scaffold N50	GC (%)	BUSCO (%)
Isolate1Pk **	34	7.2	55.9	3.8	44.8	99.2
Isolate2Pk **	29	7.8	56.5	4.1	44.8	99.6
Isolate22Pk	270	2.5	58.7	1.0	44.6	96.5
Isolate32Pk	81	3.0	57.5	1.6	44.5	95.6
Isolate36Pk	27	8.0	56.1	4.3	44. 8	97.6
Isolate42Pk	40	8.0	55.3	3.4	44.7	97.5
Isolate18Ppll	16	6.0	37.2	2.6	50.9	96.4
Isolate27Ppll	26	4.3	35.2	2.4	51.6	96.4
Isolate58Ppll	48	3.7	37.9	2.2	50.7	97.0
Isolate72Ppll	12	7.7	34.7	3.4	51.7	93.9
Isolate104Ppll	42	4.5	37.5	2.0	51.6	96.0
Isolate113Ppll	45	3.0	37.6	1.7	51.5	96.6
Isolate3Pp **	16	6.3	34.6	3.7	52.6	99.1
Isolate4Pp **	20	3.2	34.6	2.0	52.6	99.0
Isolate5Pp **	18	4.0	36.7	2.7	52.4	98.5
Isolate87Pp	14	6.2	34.5	3.8	52.5	96.6
Isolate88Pp	19	6.2	34.5	3.6	52.6	95.8
Isolate97Pp	23	4.2	35.5	2.4	52.6	95.5

** indicates isolates that have illumina reads and assembly was hybrid.

**Table 2:** Genome annotation—statistics on the number of genes identified and the BUSCO %.

	Augustus		Braker		GeneMarkES		RNAmmer	tRNAscan-SE
Isolates/Species	Gene	BUSCO (%)	Gene	BUSCO (%)	Gene	BUSCO (%)	Total rRNA	tRNA
Isolate1Pk	16,127	66.2	10,024	98.9	10,198	97.7	53	141
Isolate2Pk	14,085	73.1	9851	98.6	10,319	98.4	107	139
Isolate22Pk	15,571	60.5	10,165	84.5	10,284	89.4	75	144
Isolate32Pk	15,548	54.4	9799	83.9	9884	89.9	87	137
Isolate36Pk	12,737	69.7	9914	89.1	10,009	92.1	83	142
Isolate42Pk	9388	87.2	10,454	86.6	9717	89.8	87	128
Isolate18Ppll	12,582	89.8	13,117	90.7	12,954	91.8	59	150
Isolate27Ppll	11,090	90.2	13,014	85.9	11,503	92.5	47	137
Isolate58Ppll	14,906	70.8	10,897	88.5	11,518	92.4	133	135
Isolate72Ppll	14,156	63.1	10,746	80.5	11,056	88.3	76	135
Isolate104Ppll	11,854	88.0	14,406	91.7	12,249	90.9	40	144
Isolate113Ppll	11,594	88.6	12,217	91.4	11,988	91.4	58	135
Isolate3Pp	16,562	71.4	10,742	97.8	11,417	98.0	104	129
Isolate4Pp	11,019	97.2	10,898	97.8	11,096	94.6	55	130
Isolate5Pp	17,970	71.8	10,964	96.2	11,797	96.9	58	129
Isolate87Pp	16,693	65.2	10,872	87.3	10,953	90.1	72	129
Isolate88Pp	16,850	66.7	10,569	88.9	10,987	90.6	98	129
Isolate97Pp	11,137	87.7	12,276	85.2	11,562	91.2	54	132

**Table 3:** Read count of the expressed TE in AB species.

TE	*P. koolunga*	*P. pinodes*	*P. pinodella*
I non-LTR	7212	26,401	62,440
Mariner	326	19,131	1092
MOLLY	164	4700	3552
Gypsy	49	2954	410
Copia	89	32	1177
MarCry	18	101	105
Tad1	50	16	10
PYGGY	21	1	5
LMR1	77	1	5
AFUT1	37	0	0
REALAA	11	0	2
MGR583	6	7	7
MAGGY	1	8	0
CFT1	0	8	0
TFO1	0	2	0
POT2	0	0	2
FoHeli3	0	0	0
Total	8062	53,361	68,806

**Table 4:** Interspecies orthologous statistics showing, the total number of clusters observed across the AB species, orthogroup clusters present in at least two species, and single-copy orthogroup (SCG) clusters in AB species.

Species	Clusters	Orthologous Clusters	SCG Clusters
*P. pinodella*	11,782	3543	8239
*P. pinodes*	11,653	2438	9215
*P. koolunga*	10,305	1885	8420

**Table 5:** Annotation for unique clusters.

Species	Annotation	Protein Name
*P. pinodes*	GO:0005525	F:GTP binding
	GO:0016705	F:oxidoreductase activity, acting on paired donors, with incorporation or reduction of molecular oxygen
	GO:0004847	F:urea carboxylase activity
	Q9UV10	Heterokaryon incompatibility protein 6, OR allele
*P. pinodella*	GO:0016125	P:sterol metabolic process
	GO:0016114	P:terpenoid biosynthetic process
	GO:0055114	P:oxidation-reduction process
	GO:0005525	F:GTP binding
	GO:0006520	P:cellular amino acid metabolic process
	GO:0008643	P:carbohydrate transport
	GO:0140041	P:cellular detoxification of methylglyoxal
*P. koolunga*	GO:0009405	P:pathogenesis
	GO:0006364	P:rRNA processing
	GO:0016746	F:transferase activity, transferring acyl groups
	GO:0010124	P:phenylacetate catabolic process
	GO:0034517	P:ribophagy
	GO:0006487	P:protein N-linked glycosylation
	GO:0006810	P:transport
	GO:0004022	F:alcohol dehydrogenase (NAD) activity

**Table 6:** Mitochondrial genome assembly statistics for AB isolates.

Isolates	Species	Total Length (bp)	GC Content (%)
Isolate1Pk	*P. koolunga*	73,511	28.9
Isolate2Pk	*P. koolunga*	68,282	28.7
Isolate22Pk	*P. koolunga*	68,003	28.9
Isolate32Pk	*P. koolunga*	67,924	28.9
Isolate36Pk	*P. koolunga*	67,828	28.9
Isolate42Pk	*P. koolunga*	65,622	28.8
Isolate18Ppll	*P. pinodella*	48,429	28.8
Isolate27Ppll	*P. pinodella*	50,457	29.4
Isolate58Ppll	*P. pinodella*	49,507	29.5
Isolate72Ppll	*P. pinodella*	50,405	29.1
Isolate104Ppll	*P. pinodella*	50,009	29.5
Isolate113Ppll	*P. pinodella*	50,374	29.5
Isolate3Pp	*P. pinodes*	55,213	29.1
Isolate4Pp	*P. pinodes*	56,229	29.2
Isolate5Pp	*P. pinodes*	56,330	29.2
Isolate87Pp	*P. pinodes*	55,990	29.4
Isolate88Pp	*P. pinodes*	55,567	29.3
Isolate97Pp	*P. pinodes*	55,931	29.4

## Data Availability

The genome assemblies generated in this study is deposited in NCBI under BioProject PRJNA867522. The BioSamples accession numbers for the 18 AB isolates are SAMN30203642, SAMN30203643, SAMN30203644, SAMN30203645, SAMN30203646, SAMN30203647, SAMN30203648, SAMN30203649, SAMN30203650, SAMN30203651, SAMN30203652, SAMN30203653, SAMN30203654, SAMN30203655, SAMN30203656, SAMN30203657, SAMN30203658, and SAMN30203659.

## Supplementary Materials

The following supporting information can be downloaded at: https://www.mdpi.com/article/10.3390/jof8080884/s1, Table S1: AB species and origin; Table S2: shows raw data generated from sequencing; total read length and coverage for AB isolates.and origin; Table S3: Transcriptome sequencing statistics for AB; Table S4: Top ten repeat elements in AB species (RepeatMasker); Table S5: Mating type determination for AB species; Table S6: Numbers of core, variable, and unique genes in the three AB species using the reference isolates; Isolate2Pk, Isolate3Pp, Isolate18Ppl; Table S7: CAZyme profile of AB species; Table S8: Mitochondrial genome annotation of AB isolates using MITOS2. Pk- *P. koolunga*, pll- *P. pinodella*, pp- *P. pinodes*; Table S9: Mitochondrial annotation of AB isolates using RNAweasel. Pk- *P. koolunga*, pll- *P. pinodella*, pp- *P. pinodes*; Table S10: Mitochondrial genome intron distribution within protein coding genes among AB species. t/s: potential trans-splicing; Table S11: Mitochondrial genome codon usage in AB species; Figure S1: Blast2go top-hit species distribution for AB reference isolates a: *P. pinodes*, b: *P. pinodella* and c: *P. koolunga*; Figure S2: Dot plot comparing intraspecies AB genome assemblies aligned to its reference genomes (top 3 rows) and between the AB species (bottom); Figure S3: Repeat analysis for AB species; Figure S4: Whole genome and ortholog phylogeny of AB species; Figure S5: AB CAZyme analysis; Figure S6: Fungal cell wall degrading CAZyme analysis; Figure S7: Biosynthetic gene cluster (BCG) analysis of AB isolates; Figure S8: Fungal barcoding genes.
