# Supplementary material for: De Novo Long-Read Whole-Genome Assemblies and the Comparative Pan-Genome Analysis of Ascochyta Blight Pathogens Affecting Field Pea
Source: J Fungi (Basel). 2022 Aug 22;8(8):884. doi: 10.3390/jof8080884 (PMC9410150; doi:10.3390/jof8080884)
Supplement: Supplementary file 1 [file jof-08-00884-s001.zip › jof-1805811-supplementary.pdf]

Journal of *Fungi*

MDPI

Supplementary Materials

De Novo Long-Read Whole-Genome Assemblies and the Comparative Pan-Genome Analysis of Ascochyta Blight Pathogens Affecting Field Pea

Supplementary Table S1. AB species and origin.

Samples	S/N	ID	Year	Host	Variety	State	Site	Species
Isolate1Pk	1	GRP-15-464	2015	FieldPea	Kaspa	WA	GrassPatch	Pk
Isolate2Pk	2	PIN-15-415	2015	FieldPea	Oura	WA	Pingrup	Pk
Isolate3Pp	3	GRP-11-30	2011	FieldPea	Kaspa	WA	GrassPatch	Pp
Isolate4Pp	4	RIV-11-185	2011	FieldPea	Kaspa	SA	RivertonNVT	Pp
Isolate5Pp	5	YEL-11-87	2011	FieldPea	Kaspa	SA	Yeelanna	Pp
Isolate18Ppll	18	STH-17-057	2017	FieldPea	Gunyah	WA	Scaddan	Ppll
Isolate22Pk	22	BAL-14-264	2014	FieldPea	Kaspa	SA	BalaklavaPeaBreeding	Pk
Isolate27Ppll	27	SCD-17-092	2017	FieldPea	Wharton	WA	Scaddan	Ppll
Isolate32Pk	32	GRP-17-199	2017	FieldPea	Oura	WA	GrassPatch	Pk
Isolate36Pk	36	KIN-16-703	2016	FieldPea	Oura	SA	Kingsford	Pk
Isolate42Pk	42	RBW-11-154	2011	FieldPea	Kaspa	VIC	Rainbow	Pk
Isolate58Ppll	58	GRP-17-128	2017	FieldPea	Pearl	WA	GrassPatch	Ppll
Isolate72Ppll	72	KIN-16-675	2016	FieldPea	Kaspa	SA	Kingsford	Ppll
Isolate87Pp	87	SLK-17-353	2017	FieldPea	Pearl	VIC	SeaLake	Pp
Isolate88Pp	88	GRP-17-127	2017	FieldPea	Pearl	WA	GrassPatch	Pp
Isolate97Pp	97	BRC-11-24	2011	FieldPea	Kaspa	NSW	BoreeCreek	Pp
Isolate104Ppll	104	KAD-14-285	2014	FieldPea	Oura	SA	KadinaPeaBreading	Ppll
Isolate113Ppll	113	STH-17-065	2017	FieldPea	Gunyah	WA	South Perth	Ppll

Supplementary Table S2. Genome sequencing statistics. Table shows raw data generated from sequencing; total read length and coverage for AB isolates.

	Before pre-processing		After pre-processing	
Isolates	Total read length (bp)	Coverage (X)	Total read length (bp)	Coverage (X)
Isolate1Pk	4,982,226,679	89.2	3,704,623,294	66.33
Isolate2Pk	11,326,058,792	200.6	8,589,291,761	152.14
Isolate22Pk	2,975,195,303	50.7	2,060,794,720	35.14
Isolate32Pk	2,308,893,989	40.2	1,855,560,480	32.27
Isolate36Pk	3,846,008,904	68.6	3,109,580,221	55.46
Isolate42Pk	3,088,636,394	49.6	2,340,065,964	37.56
Isolate18Ppll	15,521,077,219	339.9	9,000,006,072	197.12
Isolate27Ppll	4,149,147,116	96.8	2,912,451,502	67.97
Isolate58Ppll	3,710,521,802	97.8	3,165,593,401	83.47
Isolate72Ppll	6,781,331,088	195.6	5,467,406,519	157.71
Isolate104Ppll	4,426,153,959	120.5	3,049,702,068	83.02
Isolate113Ppll	5,014,024,569	129.3	3,251,706,558	83.82
Isolate3Pp	6,200,516,350	179.0	3,275,416,034	94.55
Isolate4Pp	5,256,135,966	151.0	2,836,519,143	81.49
Isolate5Pp	3,055,700,906	83.4	1,853,438,494	50.57

Isolate87Pp	4,472,157,092	129.6	3,308,303,728	95.88
Isolate88Pp	6,845,192,045	198.7	4,619,910,882	134.07
Isolate97Pp	4,591,051,084	113.7	2,771,022,159	68.62

Supplementary Table S3. Transcriptome sequencing statistics for AB.

Isolates Species	Total reads before filtering	Total bases before filtering (Gb)	Total reads after filtering (Million)	Total bases after filtering (Gb)	Q20 bases (%)	GC content
Isolate1Pk	50,081,320	7.56	48.5	6.95	93.8	55.1
Isolate2Pk	17,545,006	2.65	16.9	2.42	93.8	54.8
Isolate22Pk	23,093,894	3.49	22.1	3.14	93.6	54.8
Isolate32Pk	29,174,602	4.41	27.7	4.0	93.5	54.4
Isolate36Pk	13,643,562	2.06	12.9	1.84	95.0	56.1
Isolate42Pk	6,943,954	1.05	6.4	0.87	93.2	56.0
Isolate18Ppll	18,906,428	2.85	18.3	2.63	93.9	55.5
Isolate27Ppll	31,838,402	4.81	30.8	4.40	94.0	55.5
Isolate58Ppll	24,567,250	3.71	23.2	3.31	93.2	54.1
Isolate72Ppll	17,874,650	2.70	17.1	2.47	93.6	55.7
Isolate104Ppll	34,672,678	5.24	33.6	4.78	93.9	54.9
Isolate113Ppll	30,442,572	4.60	29.4	4.19	93.8	55.6
Isolate3Pp	29,773,668	4.50	28.9	4.1	93.9	54.9
Isolate4Pp	29,047,142	4.39	28.0	4.02	93.6	55.3
Isolate5Pp	32,182,830	4.86	30.9	4.37	93.6	55.5
Isolate87Pp	28,950,658	4.37	27.9	4.05	93.6	55.5
Isolate88Pp	34,578,504	5.22	33.2	4.78	93.8	55.3
Isolate97Pp	27,744,170	4.19	26.6	3.82	93.7	54.1

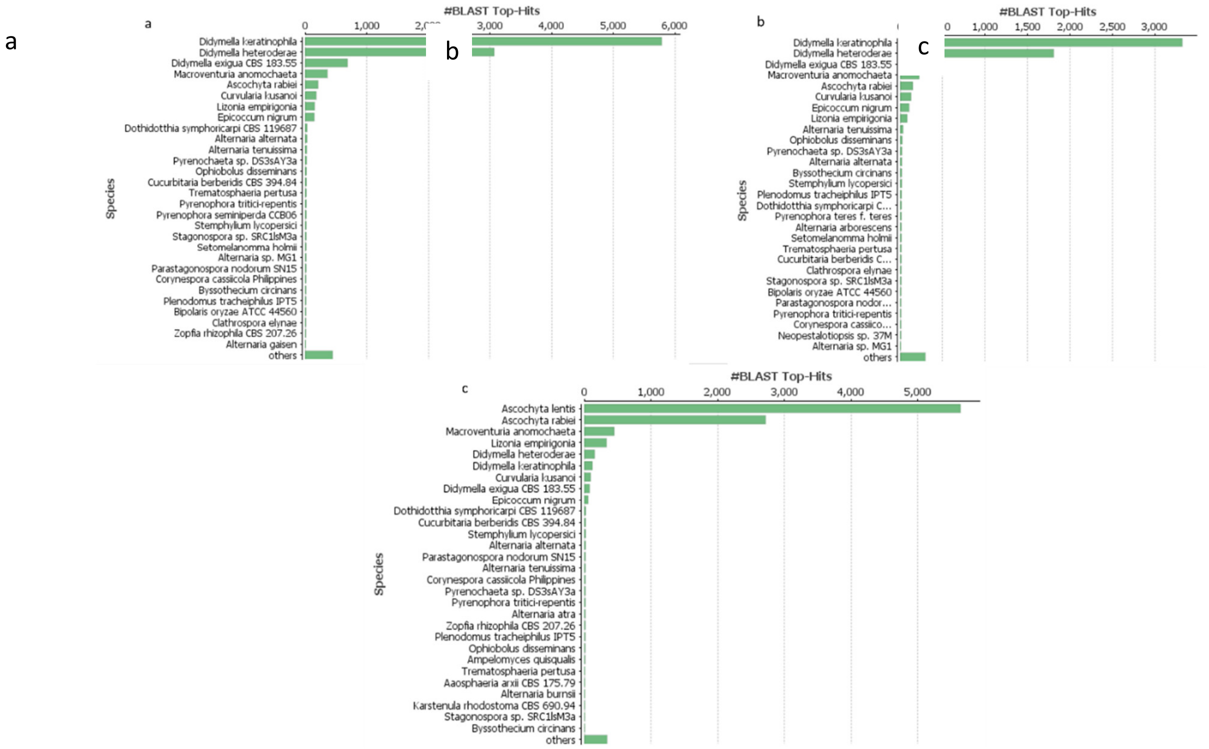

Supplementary Figure S1. Blast2go top-hit species distribution for AB reference isolates **a**: *P. pinodes*, **b**: *P. pinodella* and **c**: *P. koolunga.*

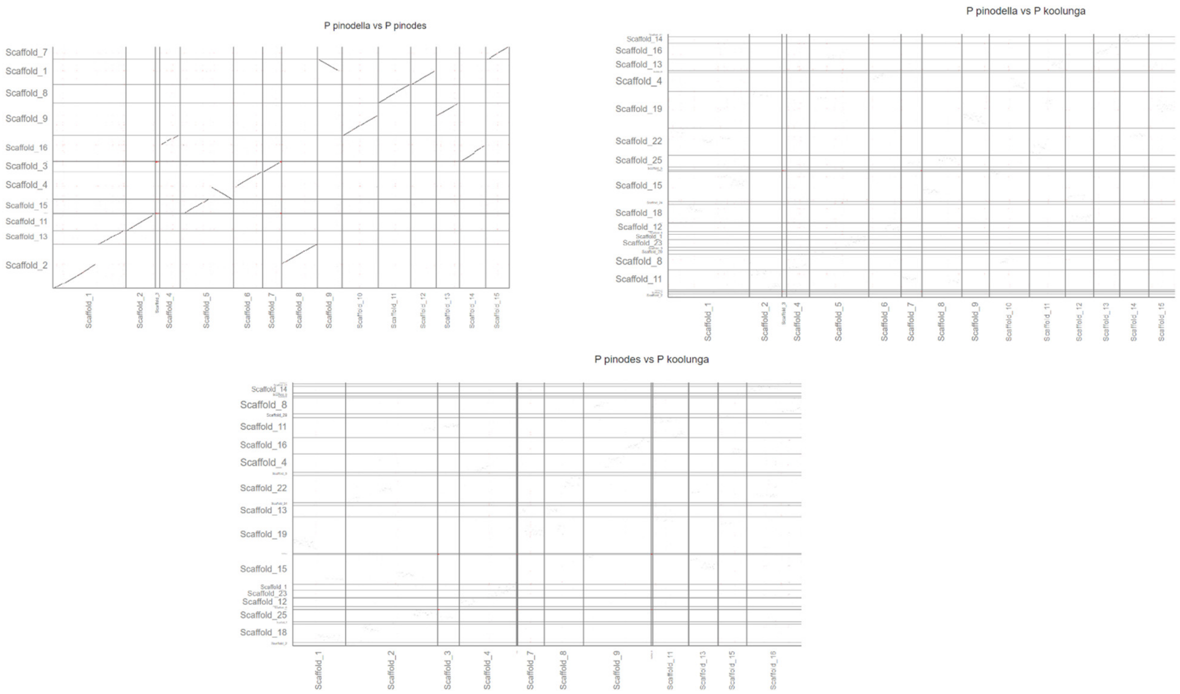

Supplementary Figure S2. Dot plot comparing intraspecies AB genome assemblies aligned to its reference genomes (top 3 rows) and between the AB species (bottom).

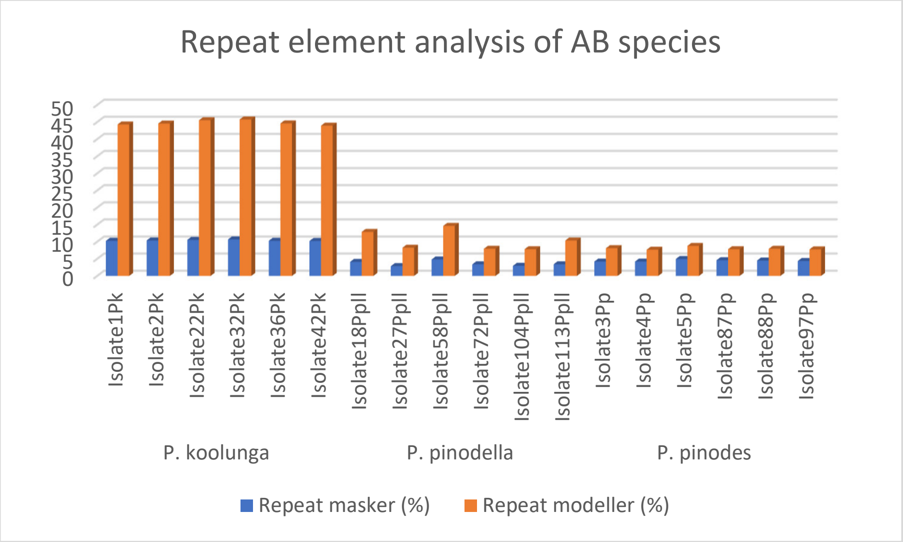

Supplementary Figure S3. Repeat analysis for AB species.

Supplementary Table S4. Top ten repeat elements in AB species (RepeatMasker).

P. koolunga	***P. pinodella***	***P. pinodes***
Simple repeats	Simple repeats	Simple repeats
GYPSY	GYPSY	GYPSY
AFUT1	MOLLY	Mariner
LMR1	Mariner	MOLLY
MOLLY	LMR1	5SrRNA
COPIA	COPIA	MarCry
Mariner	5SrRNA	Tad1
PYGGY	PYGGY	COPIA
REALAA	MarCry	LMR1
TCN-1	Tad1	I-1

Supplementary Table S5. Mating type determination for AB species: *A. lentis* MAT1-1 (DQ341314.1) and MAT1-2 (DQ341315.2) as well as *P. pinodella* MAT1-1 (JF815529.1) and MAT1-2 (JF815531.1) genes were blasted against the assembled AB genomes. *P. pinodes* and *P. pinodella* had no hits for *A. lentis* MAT1-1 and MAT1-2 but hits for *P. pinodella* MAT1-1 and MAT1-2 genes. *P. koolunga* had no hits for *P. pinodella* MAT1-1 and MAT1-2 but hits for *A. lentis* MAT1-1 and MAT1-2 genes.

**Isolates/Species**	***A. lentis* MAT1-1**	***A. lentis* MAT1-2**	***P. pinodella* MAT1-1**	***P. pinodella* MAT1-2**	**Mating gene**	**Mating pattern**
Isolate1Pk		ü			MAT1-2	self-sterile (hetero-thallic)
Isolate2Pk		ü			MAT1-2	self-sterile (hetero-thallic)
Isolate22Pk	ü				MAT1-1	self-sterile (hetero-thallic)
Isolate32Pk	ü				MAT1-1	self-sterile (hetero-thallic)
Isolate36Pk	ü				MAT1-1	self-sterile (hetero-thallic)
Isolate42Pk		ü			MAT1-2	self-sterile (hetero-thallic)

Isolate18Ppll	ü		MAT1-1	self-sterile (hetero-thallic)
Isolate27Ppll		ü	MAT1-2	self-sterile (hetero-thallic)
Isolate58Ppll	ü		MAT1-1	self-sterile (hetero-thallic)
Isolate72Ppll		ü	MAT1-2	self-sterile (hetero-thallic)
Isolate104Ppll	ü		MAT1-1	self-sterile (hetero-thallic)
Isolate113Ppll	ü		MAT1-1	self-sterile (hetero-thallic)
Isolate3Pp	ü	ü	Both	self-fertile (homo-thallic)
Isolate4Pp	ü	ü	Both	self-fertile (homo-thallic)
Isolate5Pp	ü	ü	Both	self-fertile (homo-thallic)
Isolate87Pp	ü	ü	Both	self-fertile (homo-thallic)
Isolate88Pp	ü	ü	Both	self-fertile (homo-thallic)
Isolate97Pp	ü	ü	Both	self-fertile (homo-thallic)

Supplementary Table S6. Numbers of core, variable, and unique genes in the three AB species using the reference isolates; Isolate2Pk, Isolate3Pp, Isolate18Ppll.

	P. koolunga		*P. pinodella*		*P. pinodes*	
	**Orthovenn2**	**OrthoFinder**	**Orthovenn2**	**OrthoFinder**	**Orthovenn2**	**OrthoFinder**
Core	8,721	8,479	9,916	9,781	9,757	9,625
Variable	1,607	1,601	1,835	1,951	1,867	1,594
Unique	5	3	47	62	50	43
Total	10,333	10,083	11,798	11,794	11,674	11,262

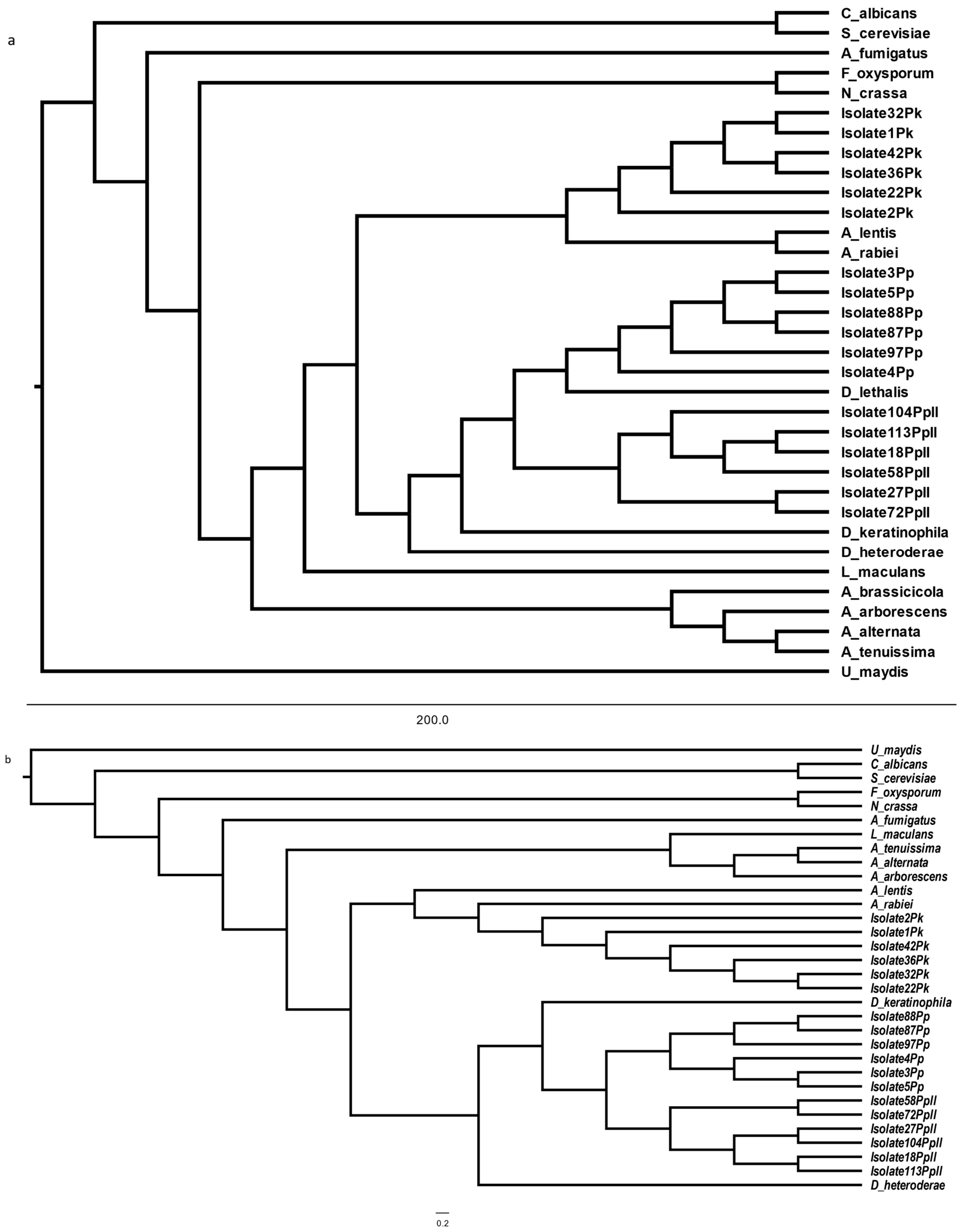
a
C_albicans
S_cerevisiae
A_fumigatus
F_oxysporum
N_crassa
Isolate32Pk
Isolate1Pk
Isolate42Pk
Isolate36Pk
Isolate22Pk
Isolate2Pk
A_lentis
A_rabiei
Isolate3Pp
Isolate5Pp
Isolate88Pp
Isolate87Pp
Isolate97Pp
Isolate4Pp
D_lethalis
Isolate104PpII
Isolate113PpII
Isolate18PpII
Isolate58PpII
Isolate27PpII
Isolate72PpII
D_keratinophila
D_heteroderae
L_maculans
A_brassicicola
A_arborescens
A_alternata
A_tenuissima
U_maydis
200.0
b
U_maydis
C_albicans
S_cerevisiae
F_oxysporum
N_crassa
A_fumigatus
L_maculans
A_tenuissima
A_alternata
A_arborescens
A_lentis
A_rabiei
Isolate2Pk
Isolate1Pk
Isolate42Pk
Isolate36Pk
Isolate32Pk
Isolate22Pk
D_keratinophila
Isolate88Pp
Isolate87Pp
Isolate97Pp
Isolate4Pp
Isolate3Pp
Isolate5Pp
Isolate58PpII
Isolate72PpII
Isolate27PpII
Isolate104PpII
Isolate18PpII
Isolate113PpII
D_heteroderae
0.2

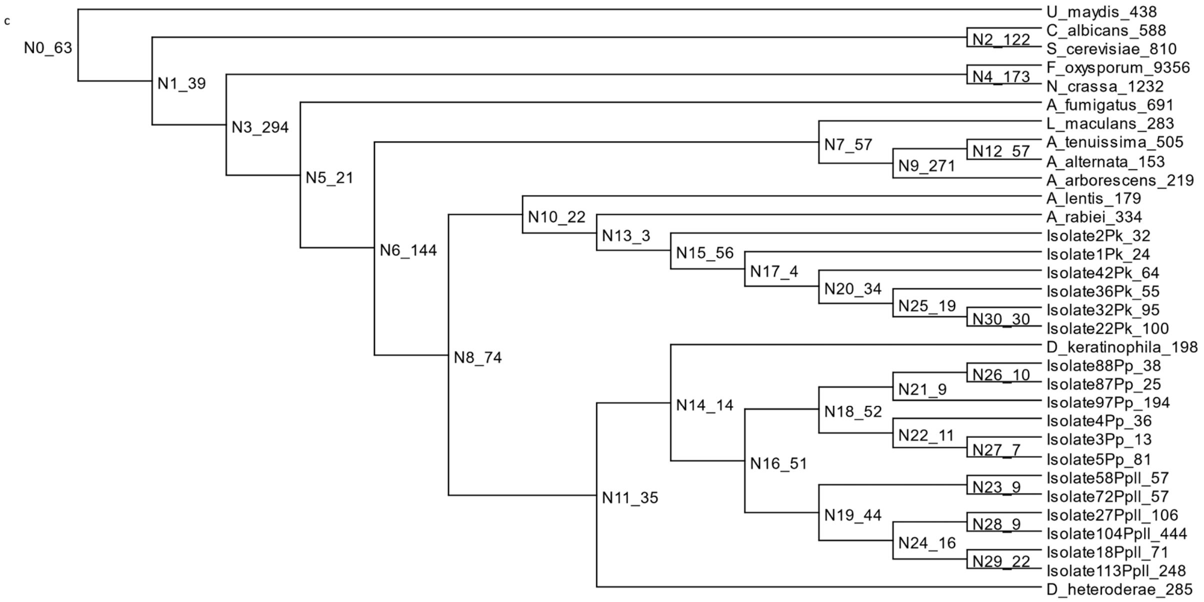

Supplementary Figure S4. Whole genome and ortholog phylogeny of AB species (**a**,**b**); NJ dendrogram using Nei's pairwise genetic distance calculation; Ortholog phylogeny of AB species showing gene duplication events (**c**).

Supplementary Table S7. CAZyme profile of AB species.

		GH	GT	PL	CE	AA	CBM	Total
P. koolunga	Isolate1Pk	239	76	32	32	71	15	465
	Isolate2Pk	242	76	32	32	72	16	470
	Isolate32Pk	238	75	31	30	70	14	458
	Isolate22Pk	241	75	31	30	69	14	460
	Isolate36Pk	236	76	33	31	69	16	461
	Isolate42Pk	235	71	33	30	74	14	457
P. pinodes	Isolate3Pp	267	78	31	32	82	20	510
	Isolate4Pp	266	73	30	35	83	17	504
	Isolate5Pp	270	80	30	33	84	21	518
	Isolate87Pp	259	73	30	33	82	17	494
	Isolate88Pp	256	76	30	32	80	18	492
	Isolate97Pp	271	74	34	34	91	15	519
P. pinodella	Isolate18Ppll	279	84	31	38	88	18	538
	Isolate27Ppll	269	79	32	36	85	17	518
	Isolate58Ppll	274	82	32	34	83	19	524
	Isolate72Ppll	272	81	31	34	79	18	515
	Isolate104Ppll	306	86	35	39	101	20	587
	Isolate113Ppll	294	81	32	40	89	20	556

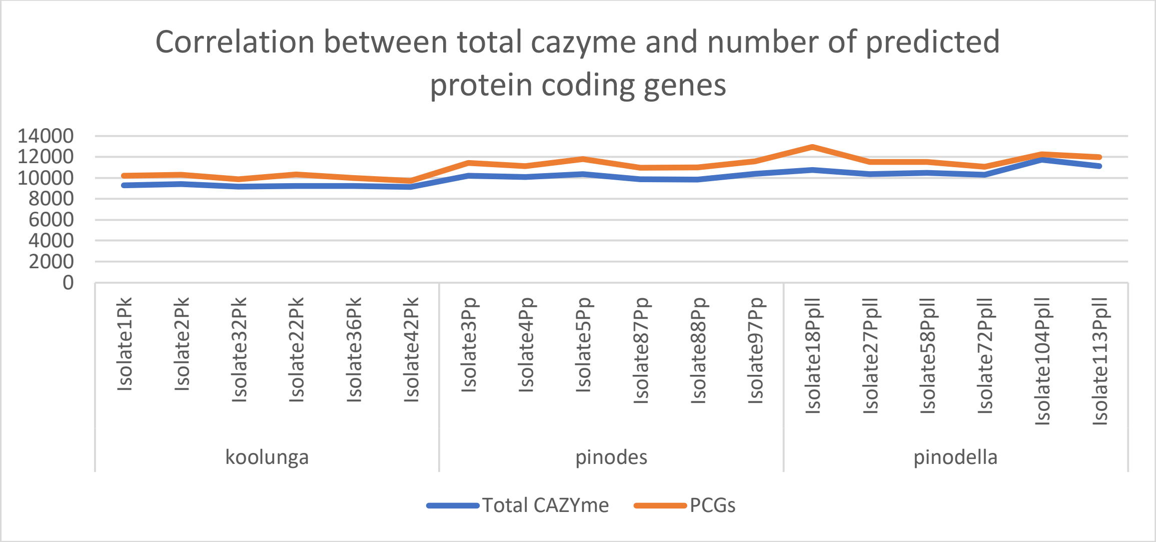

Supplementary Figure S5. AB CAZyme analysis. **a**: Correlation between the total number of CAZYmes and PCGs predicted in the genomes of AB isolates. Total number of CAZymes for each isolate was multiplied by 20 to get the numbers close enough to align the trend line with that of the number of PCG's in each isolate. **b**: CAZyme analysis of Ascomycota species.

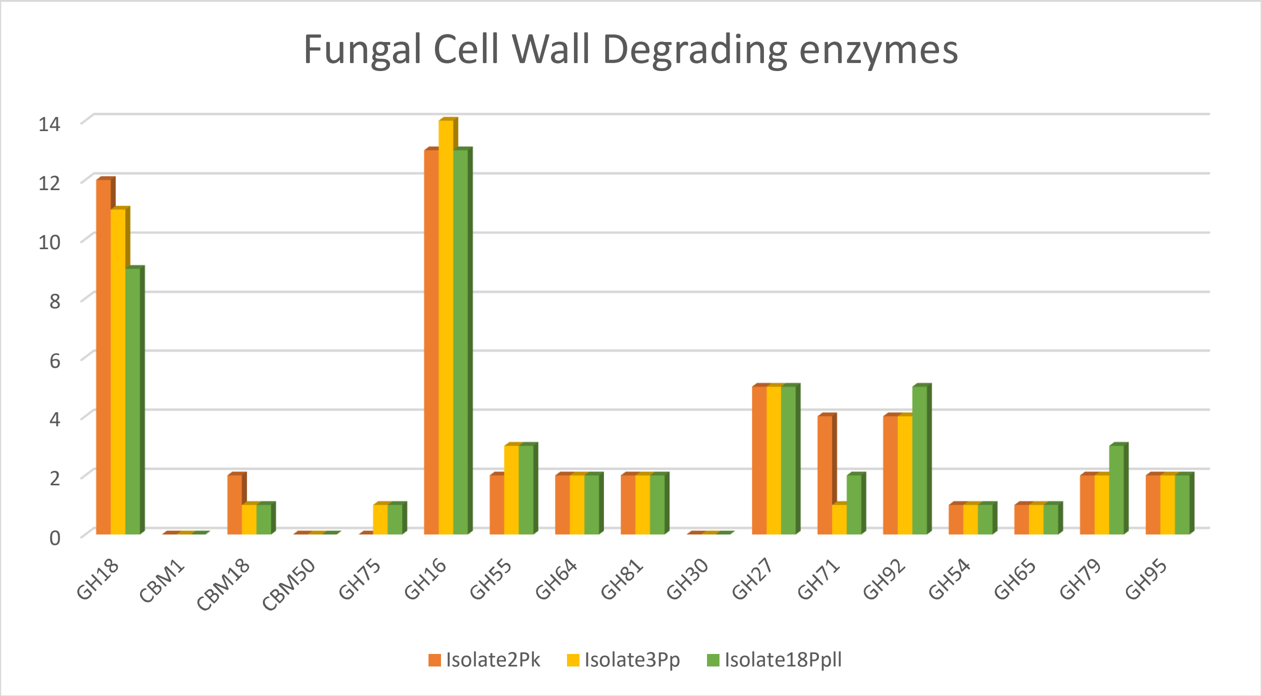

Supplementary Figure S6. Fungal cell wall degrading CAZyme analysis.

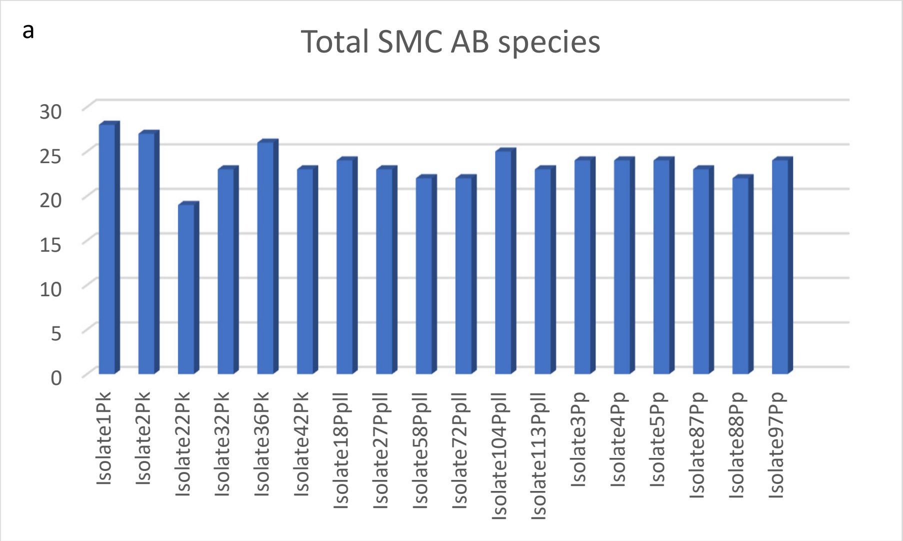
a
Total SMC AB species
30
25
20
15
10
5
0
Isolate1Pk
Isolate2Pk
Isolate22Pk
Isolate32Pk
Isolate36Pk
Isolate42Pk
Isolate18PpII
Isolate27PpII
Isolate58PpII
Isolate72PpII
Isolate104PpII
Isolate113PpII
Isolate3Pp
Isolate4Pp
Isolate5Pp
Isolate87Pp
Isolate88Pp
Isolate97Pp

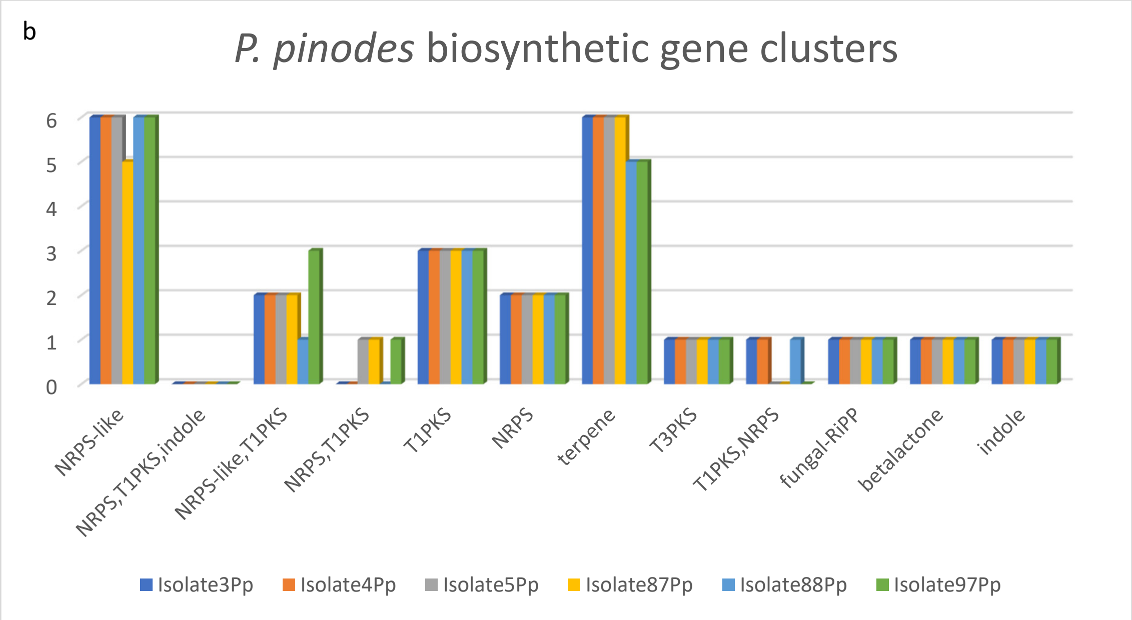
b
P. pinodes biosynthetic gene clusters
6
5
4
3
2
1
0
NRPS-like
NRPS,T1PKS,indole
NRPS-like,T1PKS
NRPS,T1PKS
T1PKS
NRPS
terpene
T3PKS
T1PKS,NRPS
fungal-RiPP
betalactone
indole
Isolate3Pp
Isolate4Pp
Isolate5Pp
Isolate87Pp
Isolate88Pp
Isolate97Pp

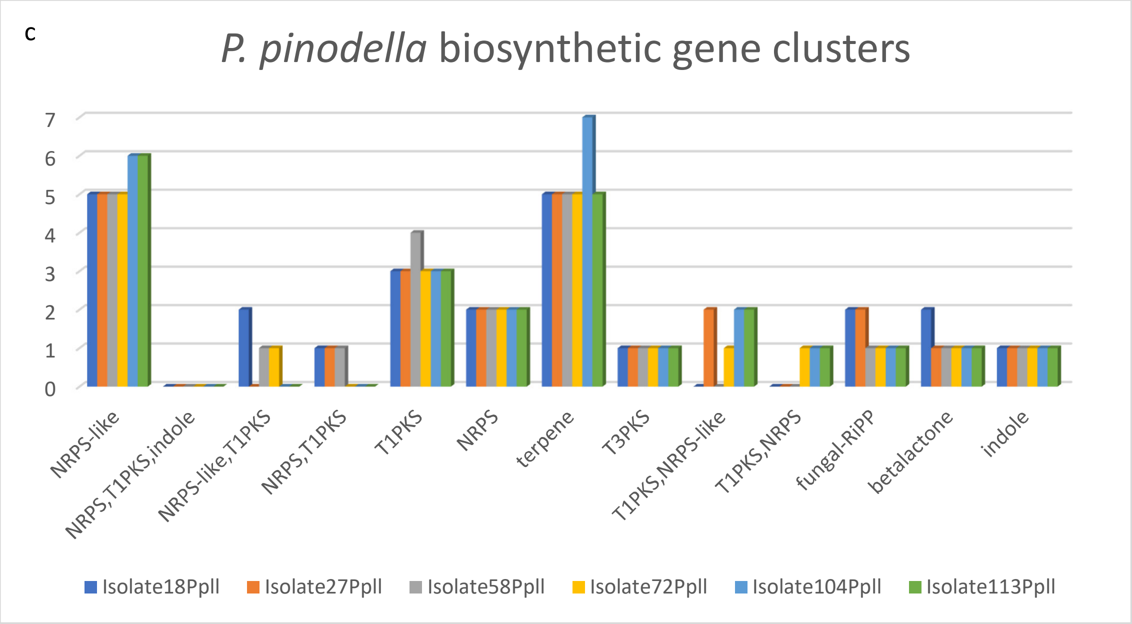

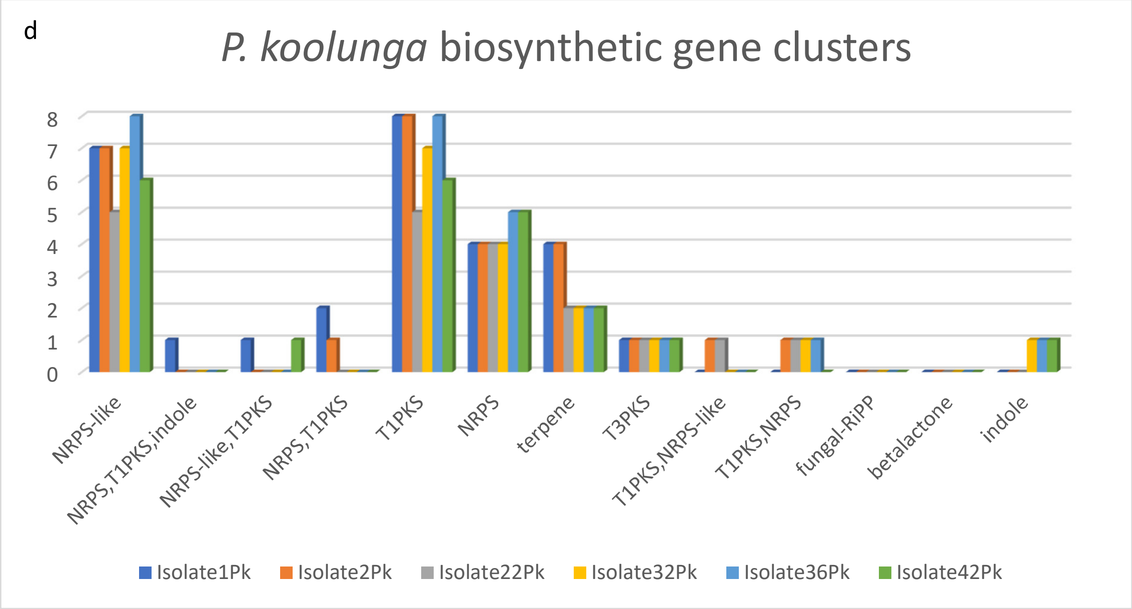

Supplementary Figure S7. Biosynthetic gene cluster (BCG) analysis of AB isolates. **a**: Total number of BCG across all AB isolates. **b**, **c** and **d**: BCG profile for each AB species.

Supplementary Table S8. Mitochondrial genome annotation of AB isolates using MITOS2. Pk- *P. koolunga*, pll- *P. pinodella*, pp- *P. pinodes.*

Isolates / Genes	1Pk	2Pk	22Pk	32 Pk	36 Pk	42 Pk	18Ppll	27Ppll	58Ppll	72Ppll	104Ppll	113 Ppll	3Pp	4Pp	5Pp	87Pp	88Pp	97Pp
cox1	8	10	8	10	7	7	10	6	6	10	7	7	7	8	12	7	8	7
cox2	3	7	6	7	6	4	4	2	3	3	4	2	3	4	3	3	2	2
cox3	4	5	4	4	4	3	4	4	4	6	5	5	4	6	5	4	6	4
cob	4	4	5	4	4	4	3	3	5	4	4	5	4	4	5	5	4	5
nad1	6	5	5	5	5	6	4	4	4	5	3	4	4	3	2	4	4	2
nad2	7	5	6	6	5	5	7	5	5	5	4	5	7	5	7	4	4	6

nad3	3	2	3	3	4	3	4	4	3	5	2	4	8	7	8	7	6	4
nad4	3	4	4	3	2	3	6	3	3	3	3	4	3	5	5	4	3	3
nad4L	4	5	5	4	5	4	4	1	1	1	1	1	1	1	1	1	1	1
nad5	5	4	3	4	3	4	11	9	10	11	8	10	13	12	12	11	12	11
nad6	3	2	2	3	2	3	2	2	2	3	2	3	3	2	1	3	2	2
rps5	1	0	1	0	0	2	0	0	0	0	0	1	0	1	1	0	0	0
rps3	5	5	5	3	6	4	4	1	2	4	3	2	2	3	2	2	2	2
rpo	2	1	1	1	1	1	0	0	0	0	0	0	0	0	0	0	0	0
lagli	39	41	35	34	39	34	16	15	13	18	11	12	25	20	18	13	14	17
giy	15	16	17	15	18	12	11	7	6	16	4	6	13	19	17	10	12	12
ATP6	1	2	1	2	2	2	3	1	1	3	1	1	2	2	2	1	2	1
rrns	1	1	1	1	1	1	1	1	1	2	1	1	1	1	1	1	1	1
rrnl	1	1	1	1	1	1	1	1	1	1	1	1	1	1	1	1	2	1

Supplementary Table S9. Mitochondrial annotation of AB isolates using RNAweasel. Pk- *P. koolunga*, pll- *P. pinodella*, pp- *P. pinodes*.

	Isolates	tRNAs without introns	rnpB (mito + plastid)	rnpB, derived (mito + plastid)	Total number of introns
P. koolunga	Isolate1Pk	27	0	0	46
	Isolate2Pk	25	0	0	45
	Isolate22Pk	27	0	0	47
	Isolate32Pk	27	0	0	47
	Isolate36Pk	27	0	0	47
	Isolate42Pk	27	0	0	47
P. pinodella	Isolate18Ppll	20	0	1	32
	Isolate27Ppll	24	0	1	37
	Isolate58Ppll	21	0	1	34
	Isolate72Ppll	25	0	1	38
	Isolate104Ppll	25	0	1	37
	Isolate113Ppll	23	0	1	36
P. pinodes	Isolate3Pp	25	1	1	39
	Isolate4Pp	25	1	1	40
	Isolate5Pp	25	1	1	40
	Isolate87Pp	24	1	1	39
	Isolate88Pp	25	1	1	40
	Isolate97Pp	24	1	1	39

Supplementary Table S10. Mitochondrial genome intron distribution within protein coding genes among AB species. t/s: potential trans-splicing.

		cob	cox1	cox2	cox3	nad1	nad2	nad4L	nad5
P. koolunga	Isolate1Pk	4	4		3	3			1
	Isolate2Pk	4	6	t/s	3	3	t/s	1	1
	Isolate22Pk	4	8	1	3	3			1
	Isolate32Pk	4	t/s	1	3	3	t/s		1
	Isolate36Pk	2	8	1	3	3			1
	Isolate42Pk	4	6	2	3	3			1

P. pinodella	Isolate18Ppll	2	4	2	2	1			1
	Isolate27Ppll	2	5	2	2	2			t/s
	Isolate58Ppll	2	5	2	2	1			1
	Isolate72Ppll	2	5	2	2	1			1
	Isolate104Ppll	2	5	2	2	1			1
	Isolate113Ppll	2	5	2	2	2			1
P. pinodes	Isolate3Pp	3	t/s	2	2	1			2
	Isolate4Pp	3	t/s	2	2	1			2
	Isolate5Pp	t/s	5	2	2	1			2
	Isolate87Pp	t/s	5	2	2	1			2
	Isolate88Pp	3	5	2	2	1			2
	Isolate97Pp	3	5	2	2	1			2

Supplementary Table S11. Mitochondrial genome codon usage in AB species.

Amino Acid	Codon	P. koolunga	P. pinodella	P. pinodes
Ala	GCG	40	33	48
	GCA	174	129	123
	GCT	271	172	228
	GCC	76	58	63
Cys	TGT	364	246	321
	TGC	158	124	135
Asp	GAT	344	242	249
	GAC	103	86	108
Glu	GAG	142	139	140
	GAA	395	255	319
Phe	TTT	1318	1024	1168
	TTC	397	288	327
Gly	GGG	99	80	91
	GGA	156	116	156
	GGT	196	162	198
	GGC	81	37	58
His	CAT	349	226	285
	CAC	150	100	131
Ile	ATA	988	678	767
	ATT	902	684	689
	ATC	346	245	281
Lys	AAG	463	318	345
	AAA	1266	969	1087
Leu	TTG	325	215	295
	TTA	1010	740	841
	CTG	198	145	178
	CTA	534	408	407
	CTT	476	337	345
	CTC	160	128	138
Met	ATG	322	282	290
Asn	AAT	915	607	642
	AAC	445	291	307
Pro	CCG	74	45	49

	CCA	208	136	148
	CCT	268	159	177
	CCC	116	66	68
Gln	CAG	192	140	180
	CAA	354	256	263
Arg	AGG	169	183	209
	AGA	472	327	307
	CGG	64	53	50
	CGA	93	66	85
	CGT	102	79	109
	CGC	64	48	35
Ser	AGT	414	275	375
	AGC	246	208	208
	TCG	72	72	83
	TCA	326	189	274
	TCT	472	316	363
	TCC	198	126	130
Thr	ACG	105	93	76
	ACA	372	326	259
	ACT	451	273	347
	ACC	246	176	175
Val	GTG	152	104	123
	GTA	449	295	399
	GTT	433	307	338
	GTC	117	76	111
Trp	TGG	154	137	165
	TGA	285	222	226
Tyr	TAT	958	723	757
	TAC	450	351	336
End	TAG	480	368	407
	TAA	1041	717	812

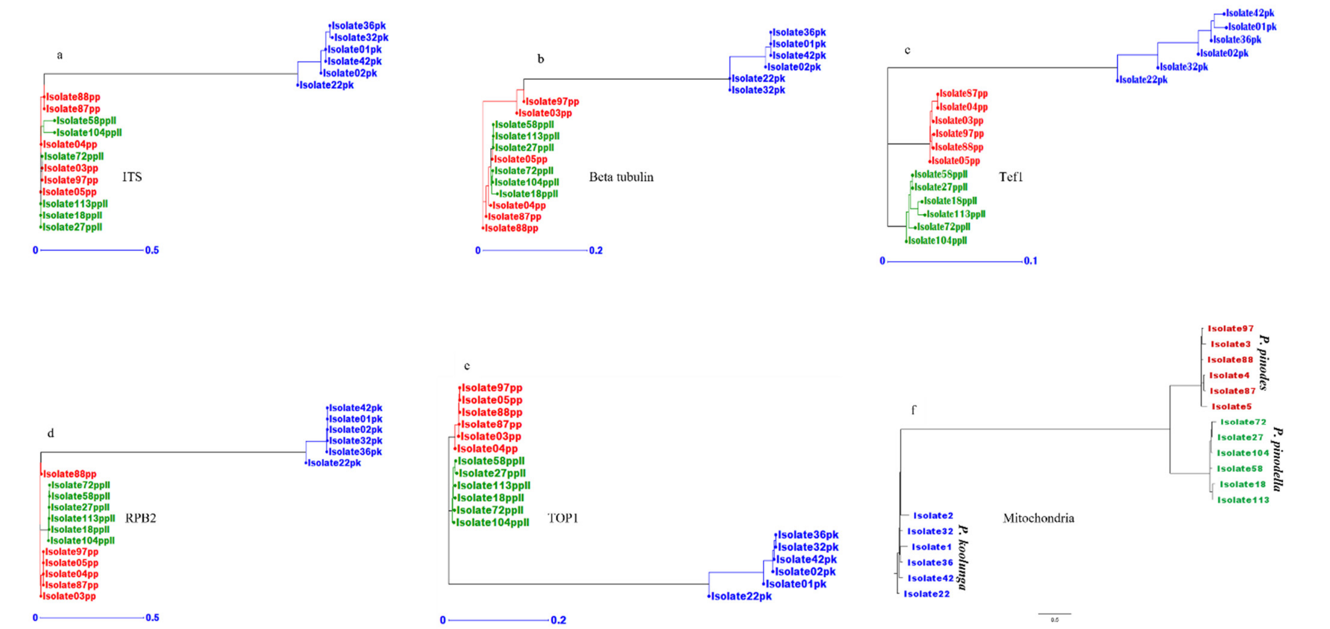

Supplementary Figure S8. Fungal barcoding genes: Relationship between AB species using ITS and protein coding genes are displayed (**a**,**b**,**c**,**d** and **e**); Parsimony phylogenetic tree of mitochondrial genome of AB species using kSNP and FigTree (**f**).
